# An Efficient *Brome mosaic virus*-Based Gene Silencing Protocol for Hexaploid Wheat (*Triticum aestivum* L.)

**DOI:** 10.3389/fpls.2021.685187

**Published:** 2021-06-18

**Authors:** Yongqin Wang, Chenglin Chai, Behnam Khatabi, Wolf-Rüdiger Scheible, Michael K. Udvardi, Malay C. Saha, Yun Kang, Richard S. Nelson

**Affiliations:** Noble Research Institute, LLC, Ardmore, OK, United States

**Keywords:** *Brome mosaic virus*, virus-induced gene silencing, BMV-VIGS, insert stability, Wheat (*Triticum aestivum*), *PHYTOENE DESATURASE*, *PHOSPHATE2*, functional genomics

## Abstract

Virus-induced gene silencing (VIGS) is a rapid and powerful method to evaluate gene function, especially for species like hexaploid wheat that have large, redundant genomes and are difficult and time-consuming to transform. The *Brome mosaic virus* (BMV)-based VIGS vector is widely used in monocotyledonous species but not wheat. Here we report the establishment of a simple and effective VIGS procedure in bread wheat using BMVCP5, the most recently improved BMV silencing vector, and wheat genes *PHYTOENE DESATURASE* (*TaPDS*) and *PHOSPHATE2* (*TaPHO2*) as targets. Time-course experiments revealed that smaller inserts (~100 nucleotides, nt) were more stable in BMVCP5 and conferred higher silencing efficiency and longer silencing duration, compared with larger inserts. When using a 100-nt insert and a novel coleoptile inoculation method, BMVCP5 induced extensive silencing of *TaPDS* transcript and a visible bleaching phenotype in the 2nd to 5th systemically-infected leaves from nine to at least 28 days post inoculation (dpi). For *TaPHO2*, the ability of BMVCP5 to simultaneously silence all three homoeologs was demonstrated. To investigate the feasibility of BMV VIGS in wheat roots, ectopically expressed *enhanced GREEN FLUORESCENT PROTEIN* (*eGFP*) in a transgenic wheat line was targeted for silencing. Silencing of eGFP fluorescence was observed in both the maturation and elongation zones of roots. BMVCP5 mediated significant silencing of *eGFP* and *TaPHO2* mRNA expression in roots at 14 and 21 dpi, and *TaPHO2* silencing led to the doubling of inorganic phosphate concentration in the 2nd through 4th systemic leaves. All 54 wheat cultivars screened were susceptible to BMV infection. BMVCP5-mediated *TaPDS* silencing resulted in the expected bleaching phenotype in all eight cultivars examined, and decreased *TaPDS* transcript was detected in all three cultivars examined. This BMVCP5 VIGS technology may serve as a rapid and effective functional genomics tool for high-throughput gene function studies in aerial and root tissues and in many wheat cultivars.

## Introduction

Bread wheat (*Triticum aestivum* L.) is the second largest food crop in the world by acreage (USDA ERS., 2018). Biotic and abiotic stresses, such as rust pathogens and drought (Ellis et al., 2014; Lesk et al., 2016; Yu et al., 2018; Babu et al., 2020), and deficiency or poor use of nutrients, particularly nitrogen and phosphate (López-Arredondo et al., 2014; Chen and Liao, 2017; Hawkesford, 2017), limit wheat production. The dramatic increase in global wheat production during the past several decades was mainly due to the adoption of high-yielding and input-responsive wheat varieties (Tadesse et al., 2016). Continued improvement in wheat production by developing broadly adapted, abiotic and biotic stress tolerant, nutrient and water efficient, and high yielding varieties requires the continued utilization of modern breeding and biotechnological approaches.

Bread wheat has a large and complex genome (~17 Gb, ~129,000 genes, with 80–90% repetitive sequences), and significant genome variation (~36% or ~47,000 genes) exists among cultivars (Wanjugi et al., 2009; Safar et al., 2010; Montenegro et al., 2017). Being an allohexaploid, bread wheat contains three diploid sets of seven chromosomes each from three parents (2n = 6x = 42, genomes AABBDD) (Feldman and Levy, 2012). In the Chinese Spring reference genome, more than half of the high-confidence protein-coding genes are present as exactly three homoeologous copies (one copy per subgenome A, B, and D), and homoeologs on average share over 95% similarity within their coding regions (Adamski et al., 2020). Transcriptional analysis revealed that 70% of the three homoeologous copies show balanced and ubiquitous expression (Ramírez-González et al., 2018). Functional redundancy between homoeologs is commonly observed in wheat due to the high similarity in sequence and expression pattern among homoeologs (Uauy, 2017). The increasing availability of genetic and genomic resources, transcriptomic platforms and novel biotechnology approaches greatly facilitate our understanding of gene function in this species (Jia et al., 2018). However, functional redundancy between homoeologs makes it difficult to perform functional genomics research in wheat using stable genetics or mutant resources. Extended time is required to identify plants with all homoeologs modified even with cultivars having more rapid seeding cycles and new biotechnology techniques such as genome-editing allowing analysis in the first generation of stable transformants (Zhao et al., 2016; Liang et al., 2017, 2018; Zong et al., 2017; Adamski et al., 2020).

Virus-induced gene silencing (VIGS), a transient RNA-silencing technology that exploits the plant antiviral defense system (Baulcombe, 1999; Vance and Vaucheret, 2001; Voinnet, 2001), could overcome many of the difficulties associated with other procedures for studying gene function in this species. To initiate VIGS, a host gene fragment is expressed through the viral vector during infection of the host plant, which triggers the accumulation of virus-derived small interfering RNAs (siRNAs) and consequently leads to sequence-specific degradation of both the viral RNA and the mRNA transcribed from the host target gene (Baulcombe, 2004; Csorba et al., 2009). VIGS is capable of silencing a single gene, a subset of or all homoeologs, or even a gene family. As an alternative to stable transformation to knock down gene expression, VIGS provides a simple, rapid, inexpensive and effective way for gene function characterization in plants. It can be used for high-throughput prescreening of candidate genes identified through genome-wide association studies or RNA-sequencing analysis before using more definitive but also more time-consuming gene function analyses (Scofield and Nelson, 2009; Adamski et al., 2020).

In the past two decades, over 50 plant VIGS vectors have been generated (Dommes et al., 2019). However, most VIGS vectors functioning in eudicots are not effective in monocots (Bekele et al., 2019; Dommes et al., 2019; Kant and Dasgupta, 2019). Among those developed for monocotyledons, three have been applied to silence genes in wheat. *Barley stripe mosaic virus* (BSMV) has been extensively employed to assess gene function in wheat, as well as other *Triticeae* species (Dommes et al., 2019; Li et al., 2020; Zhou et al., 2020). The recently developed *Foxtail mosaic virus* (FoMV)-based VIGS system triggered efficient gene silencing in a broad range of host plants, including wheat (Liu et al., 2016; Mei et al., 2016). A modified *Chinese wheat mosaic virus* (CWMV) mediated efficient silencing of wheat genes at low temperature (Yang et al., 2018). Selection of a particular VIGS vector for use is influenced by the biosafety laws of the country and institution where the interested researcher resides and by any previous history the researcher may have with a particular vector. Therefore, it is worthwhile to introduce additional vector options that may better fulfill researcher and biosafety requirements.

*Brome mosaic virus* (BMV) is a popular VIGS vector for monocotyledonous species such as maize, sorghum, rice, barley, and tall fescue (Singh et al., 2018; Dommes et al., 2019; Jiao et al., 2021). Recently, an improved BMV VIGS vector, BMVCP5, displayed greater gene insert stability and enhanced target gene silencing in maize, compared with its older version (Ding et al., 2018). Although wheat is a natural host of BMV (Mise and Pocsai, 2004; Hodge et al., 2019), no BMV VIGS studies have been reported in wheat. To date, most BMV-mediated VIGS studies analyzed gene function in leaves (Pacak et al., 2010a; van der Linde et al., 2011; Sun et al., 2013; Ding et al., 2018), and one study in sorghum showed genes in the inflorescence could be silenced (Singh et al., 2018). The ability of this BMV vector to silence gene expression in other tissues has not been reported. In this study, we chose wheat homologs of *PHYTOENE DESATURASE* (*PDS*) and *PHOSPHATE2* (*PHO2*) as targets to evaluate the feasibility of BMV VIGS system in wheat leaves and roots. *PDS*, a key enzyme for the synthesis of β-carotenoids necessary for photoprotection among other functions (Ruiz-Sola and Rodríguez-Concepción, 2012), has been targeted in many VIGS systems due to the easily observed bleaching phenotype on leaves associated with its silencing (Yuan et al., 2011; Liu et al., 2016; Yang et al., 2018). *PHO2*, encoding an ubiquitin-conjugating E2 enzyme, negatively regulates inorganic phosphate (Pi) uptake and root-to-shoot translocation (Aung et al., 2006; Bari et al., 2006). In reciprocal grafting experiments with Arabidopsis, a *pho2 (PHO2* loss of function mutant) genotype root resulted in increased Pi accumulation in grafted wild-type (normal *PHO2*) shoot, and a wild-type root is sufficient to restore wild-type levels of Pi in the grafted *pho2* genotype shoot (Bari et al., 2006). Findings from that study indicate that *PHO2* may serve as a target to study VIGS activity in roots, and indeed, BSMV-mediated silencing of *PHO2* homologs in barley resulted in the expected Pi accumulation in leaves (Pacak et al., 2010b).

Here we report that BMVCP5 silencing vector, targeting *TaPDS* and *TaPHO2* or *eGFP*, functions in leaves, shoots and roots, as well as in multiple hexaploid wheat cultivars. Additionally, we investigated the optimal insert size for this vector to maintain gene silencing in wheat.

## Results

### A Simple and Efficient Method for BMV-Based VIGS in Wheat

**Figure 1:**
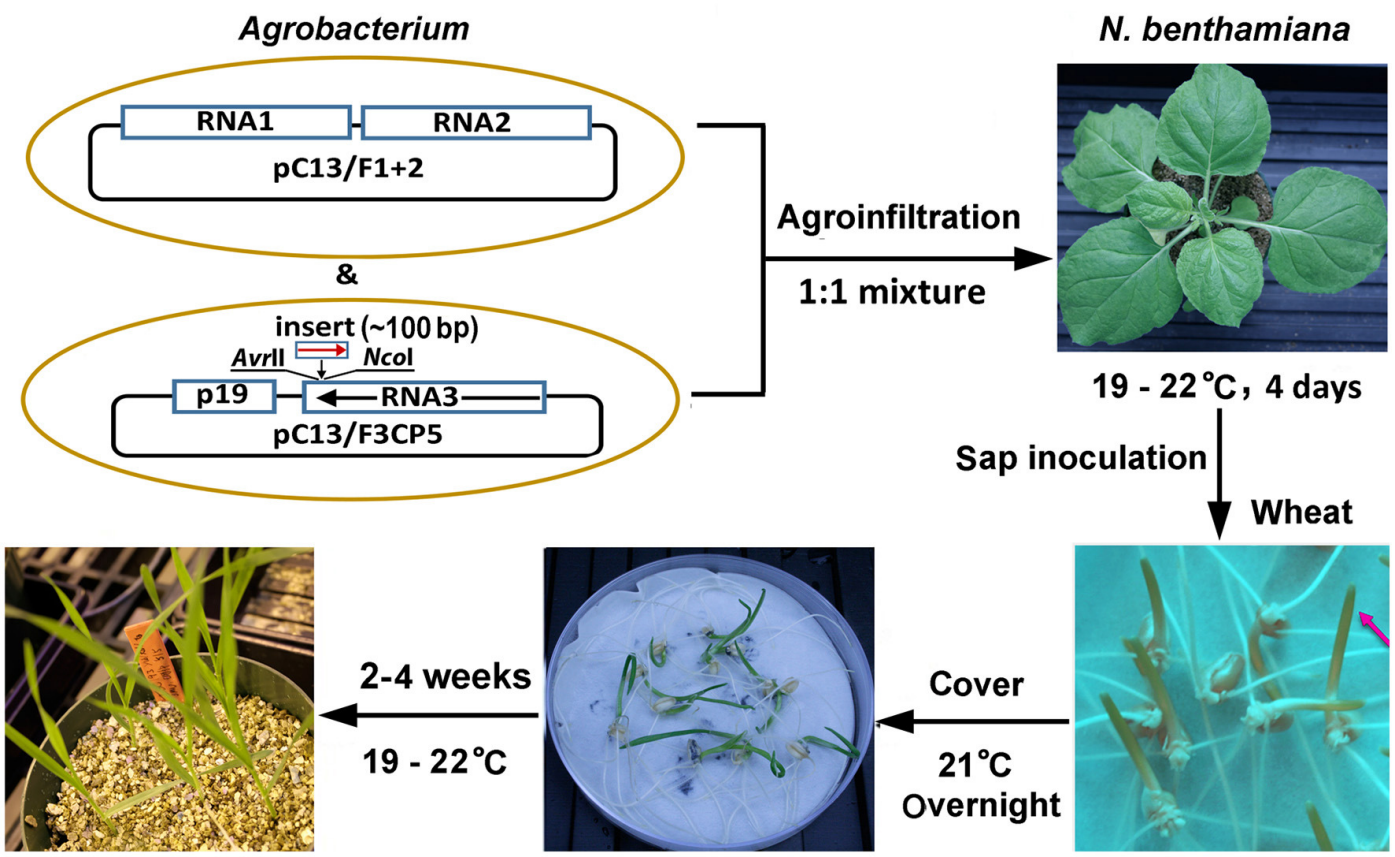
Protocol for BMVCP5-mediated gene silencing in wheat. Binary vectors pC13/F1 + 2 and pC13/F3CP5 contain sequences allowing expression of RNAs 1 and 2 and RNA 3, respectively, of the BMVCP5 silencing vector. For VIGS, a host gene fragment, optimally of ~100-bp, is inserted into the *Nco*I and *Avr*II sites in RNA3 sequence of pC13/F3CP5 in an antisense orientation. *Agrobacterium* containing the binary vectors with BMVCP5 are infiltrated into *Nicotiana benthamiana* leaves for multiplication of the viral silencing vector. *N. benthamiana* leaves are harvested at 4 days post infiltration, and crude sap is prepared for mechanical (rub) inoculation to wheat coleoptiles at 3 days post germination (or wheat leaves at 7–9 days post germination). Red arrow points to coleoptile for inoculation. Inoculated wheat seedlings are covered, kept at room temperature (21°C) overnight, and then transferred to solid media (or a hydroponic growth system) and grown in a greenhouse at 19–22°C for 2–4 weeks for target gene silencing.

BMV is a tripartite RNA virus with a genome comprising three positive-sense RNAs, RNAs 1–3 (Noueiry and Ahlquist, 2003). The improved BMV-based two-part silencing vector, BMVCP5, is *Agrobacterium*-based with RNAs 1 and 2 expressed from binary plasmid pC13/F1 + 2, and RNA 3 expressed from binary plasmid pC13/F3CP5 (Ding et al., 2018). Containing modifications from the previous BMV VIGS protocols for maize and sorghum (Zhu et al., 2014; Ding et al., 2018; Singh et al., 2018) and optimized based on the findings from this study (see below and discussion), a simple and effective BMVCP5 VIGS procedure was established in wheat (Figure 1). Major steps of this protocol include: (1) construction of the BMV VIGS vector by inserting a target gene fragment of ~100 base pair (bp) into the *Nco*I and *Avr*II sites, located in the 3′ UTR of RNA3 immediately after the stop codon of the coat protein, of pC13/F3CP5 in an antisense orientation; (2) amplification of the VIGS vector in *Nicotiana benthamiana* leaves via agro-infiltration; (3) rub-inoculation of coleoptiles of 3 day-old or leaf blades of 7–9 day-old wheat seedlings using crude sap from infected *N. benthamiana* leaves; and (4) growing the inoculated wheat plants at 19–22°C for 2–4 weeks before silencing characterization.

### Determining Optimal Insert Size for BMVCP5 VIGS in Wheat

*TaPDS* and *TaPHO2* were chosen as silencing targets for proof-of-concept studies to determine the feasibility of BMV VIGS system in hexaploid winter wheat cultivar (*cv*.) Overley. To ensure only the three homoeologs of *TaPDS* or *TaPHO2* were targeted, insert sequences were chosen at a highly specific and conserved region within the target genes. Different size inserts for the same target gene overlapped with each other to share as much common sequence as possible (Supplementary Figure 1). The stability of *TaPDS, TaPHO2*, and *eGFP* fragments varying in size from 100 to 252 nt in the BMV vector was evaluated. Vectors carrying *eGFP* fragments, which have no target in the wheat genome, were used as a non-silencing vector control in VIGS studies targeting *TaPDS* or *TaPHO2*. Such a control, as opposed to one without an insert, would produce an infection likely more similar in virus accumulation and disease effect on the host to those induced by the viruses inserted with fragments of target genes (Zhu et al., 2014).

**Figure 2:**
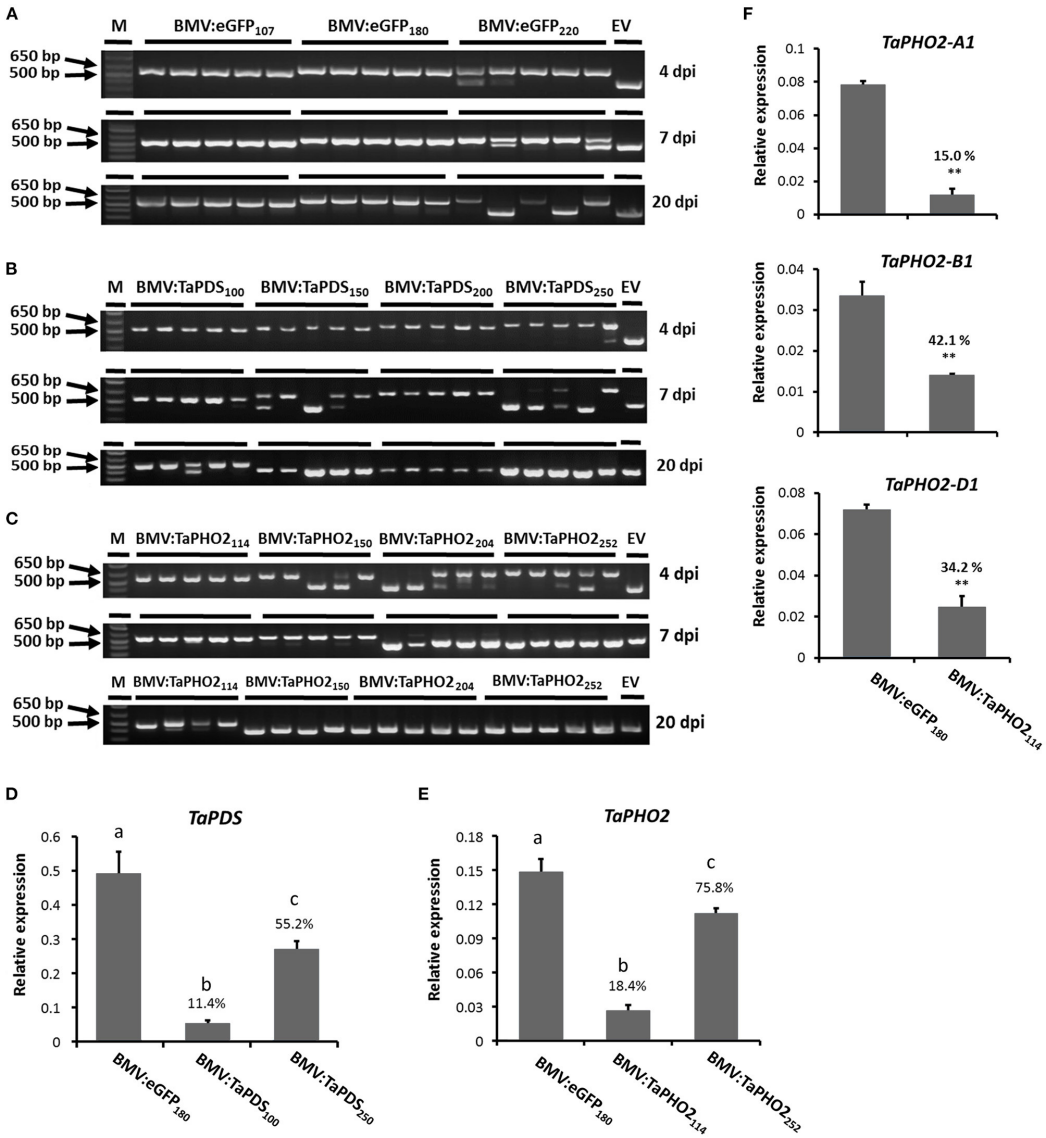
Stability of insert fragments and gene silencing in wheat leaves with BMVCP5. RT-PCR products from wheat leaves of plants inoculated with BMVCP5 harboring gene fragments in length from 100 to 252 nt from *eGFP* **(A)**, *TaPDS* **(B)**, and *TaPHO2* **(C)**. Subscript numbers after target gene designation indicate the insert size in the BMV vector. The 1st or 2nd inoculated leaf or 3rd systemic leaf were harvested, respectively, at 4, 7, and 20 dpi, and total RNA extracted for RT-PCR amplification using primers flanking the cloning site (P4-F/P4-R). Each lane represents an RT-PCR product from an individual plant. Similar results were obtained in three independent experiments. A 1 kb plus DNA ladder (M, Invitrogen) and a 427-bp PCR product amplified from plasmid pC13/F3CP5 with no insert (EV) serve as size markers. **(D–F)** Relative expression levels of target gene mRNA in the 3rd systemic leaf at 20 dpi were determined by RT-qPCR using the same cDNA analyzed for insert stability in Panels **(A–C)** and primers specific for the host target mRNA (but not the relevant insert fragment). PCR product quantities for target genes were normalized against the levels of wheat translation elongation factor subunit EF1α (*TaEF1*α) mRNA. **(D)** Relative expression levels of *TaPDS* mRNA in plants infected with BMV:eGFP_180_, BMV:TaPDS_100_ or BMV:TaPDS_250_. **(E)** Relative expression levels of *TaPHO2* mRNA in plants infected with BMV:eGFP_180_, BMV:TaPHO2_114_ or BMV:TaPHO2_252_. **(F)** Relative expression levels of *TaPHO2-A1, TaPHO2-B1, TaPHO2-D1* mRNA in plants infected with BMV:eGFP_180_ or BMV:TaPHO2_114_. Values in panels **(D–F)** represent means + SE of four or five biological replicates. Significant differences between treatment mean values in panels **(D,E)** are indicated by different letters above bars for each treatment (*P* < 0.05, significant ANOVA followed by LSD analysis); treatment values in panel **(F)** were compared by *t*-test (**P < 0.01). Percentage values are relative to the BMV:eGFP_180_ control.

Before inoculating the wheat seedlings, the stabilities of the inserts in the virus vector in *N. benthamiana* sap were analyzed by reverse transcription polymerase chain reaction (RT-PCR), using primers flanking the insert location. All gene fragment inserts were stably maintained in the BMVCP5 silencing vector extracted from *N. benthamiana* leaves at 4 days post infiltration except for a minor insert loss from the 252-nt *TaPHO2* insert (Supplementary Figure 2). *N. benthamiana* leaf sap containing the BMV vector progeny was then rub-inoculated on the first two leaves of 9 day-old wheat seedlings. Analysis of insert stability in infected wheat demonstrated that longer inserts were more rapidly lost than shorter inserts (Figure 2). For *eGFP*, the 220-nt insert was partially lost in the 1st inoculated leaf at 4 dpi in 1 out of 5 plants, in the 2nd inoculated leaf at 7 dpi in 2 out of 5 plants, and completely lost in the 3rd systemic leaf above the inoculated leaves at 20 dpi in 2 out of 5 plants (Figure 2A). The 107- and 180-nt fragments were maintained in all leaves analyzed at all dpi. For *TaPDS*, the 250-nt insert was partially lost in 1 out of 5 plants but shorter inserts remained intact in the 1st inoculated leaf at 4 dpi (Figure 2B). In the 2nd inoculated leaf at 7 dpi, the 100- and 200-nt inserts were stable in all 5 plants, while the 150- and 250-nt inserts were partially or completely lost in over half the plants. Lastly, in the 3rd systemic leaf at 20 dpi, all the 150-, 200-, and 250-nt inserts were completely lost, but the 100-nt insert was still intact in most of the infected plants. Similar results were obtained with *TaPHO2*, where the 114-nt insert was more stable than the 150-, 204-, and 252-nt inserts (Figure 2C). Taken together, shorter gene fragment inserts (~100 nt) were generally more stable than longer inserts in BMVCP5 during infection of wheat leaves.

Next, to compare the silencing effects induced by the smallest and largest inserts, transcript abundance of *TaPDS* and *TaPHO2* in the 3rd systemic leaf at 20 dpi from plants inoculated with BMVCP5 vectors containing the 100- and 250-nt *TaPDS* fragments and the 114- and 252-nt *TaPHO2* fragments was investigated by quantitative reverse transcription PCR (RT-qPCR). Samples from plants inoculated with BMV:eGFP_180_ were analyzed as a non-silencing control. Primers simultaneously amplifying all three homoeologs of *TaPDS* or *TaPHO2* were synthesized. Transcripts representing the *TaPDS* homoeologs were greatly down-regulated in plants infected with BMV:TaPDS_100_: an 89% decrease on average compared with the control (Figure 2D). Interestingly, even though the BMV:TaPDS_250_ vector had completely lost its insert in the 3rd systemic leaves (Figure 2B), *TaPDS* transcripts were knocked down to 55% of the BMV:eGFP_180_ control. Similarly, a large decrease in *TaPHO2* transcripts was observed in the 3rd systemic leaf at 20 dpi infected with BMV:TaPHO2_114_ (81%), but not with BMV:TaPHO2_252_ (24%; Figure 2E). In summary, BMVCP5 with a smaller insert, 100-nt for *TaPDS* or 114-nt for *TaPHO2*, induced greater silencing of the target gene than virus with a larger insert, 250-nt *TaPDS* or 252-nt *TaPHO2*. These data show that insert length was inversely related to the insert stability and target transcript silencing with the BMVCP5 VIGS system in wheat, and an optimal insert size for this vector is around 100 nt.

Although the 114-nt *TaPHO2* insert had 100% identity with each of the three target homoeologs (Supplementary Figure 1), we wanted to verify that transcript from each homoeolog was downregulated. RT-qPCR analysis was performed using homoeolog-specific primers (representing *TaPHO2-A1, -B1*, and *-D1*) and the same cDNA templates that were used for analysis of the overall *TaPHO2* expression in Figure 2E. All three homoeologs were silenced in this tissue but at different levels, with expression of *TaPHO2-A1*, -*B1*, and *-D1* decreased to 15, 42, and 34%, respectively, relative to the values from BMV:eGFP_180_-infected control tissue (Figure 2F).

### BMVCP5-Mediated *TaPDS* Silencing Features in Wheat Leaves

Analysis of the target gene silencing efficiency (amount of visible silencing per leaf) and effectiveness (number and position of leaves showing silencing) mediated by the BMVCP5 VIGS system helps determine its usefulness for functional studies in wheat (see Senthil-Kumar and Mysore, 2011 and Dommes et al., 2019 for further description of these silencing characteristic categories). These characteristics were analyzed in systemic leaves during plant development using *TaPDS* as the target. In wheat seedlings inoculated with BMV:TaPDS_100_ via the leaf blade rub-inoculation method, longitudinal photo-bleaching patches often appeared on the 2nd through 5th systemic leaves where virus disease symptoms often developed (Supplementary Figure 3A). The white bleaching phenotype associated with *TaPDS* silencing was clearly distinct from the yellow chlorosis phenotype in plants infected with BMV:eGFP_180_. Significant silencing of *TaPDS* genes in the 3rd systemic leaves at 20 dpi (Figure 2D), 4th systemic leaves at 28 dpi and 5th systemic leaves at 35 dpi were detected at the transcript level (Supplementary Figure 3B). Insert stability analysis revealed that the majority of virus vectors maintained their full-length insert, regardless of gene source, at 28 dpi, but by 35 dpi the majority of leaves contained virus that had lost some or all of the respective gene fragment inserts (Supplementary Figure 3C). By contrast, photobleaching caused by the 250-nt insert more often occurred in the 3rd and 4th systemic leaves, appeared more intense (whiter) than that induced by the 100-nt insert, and did not show in succeeding leaves.

**Figure 3:**
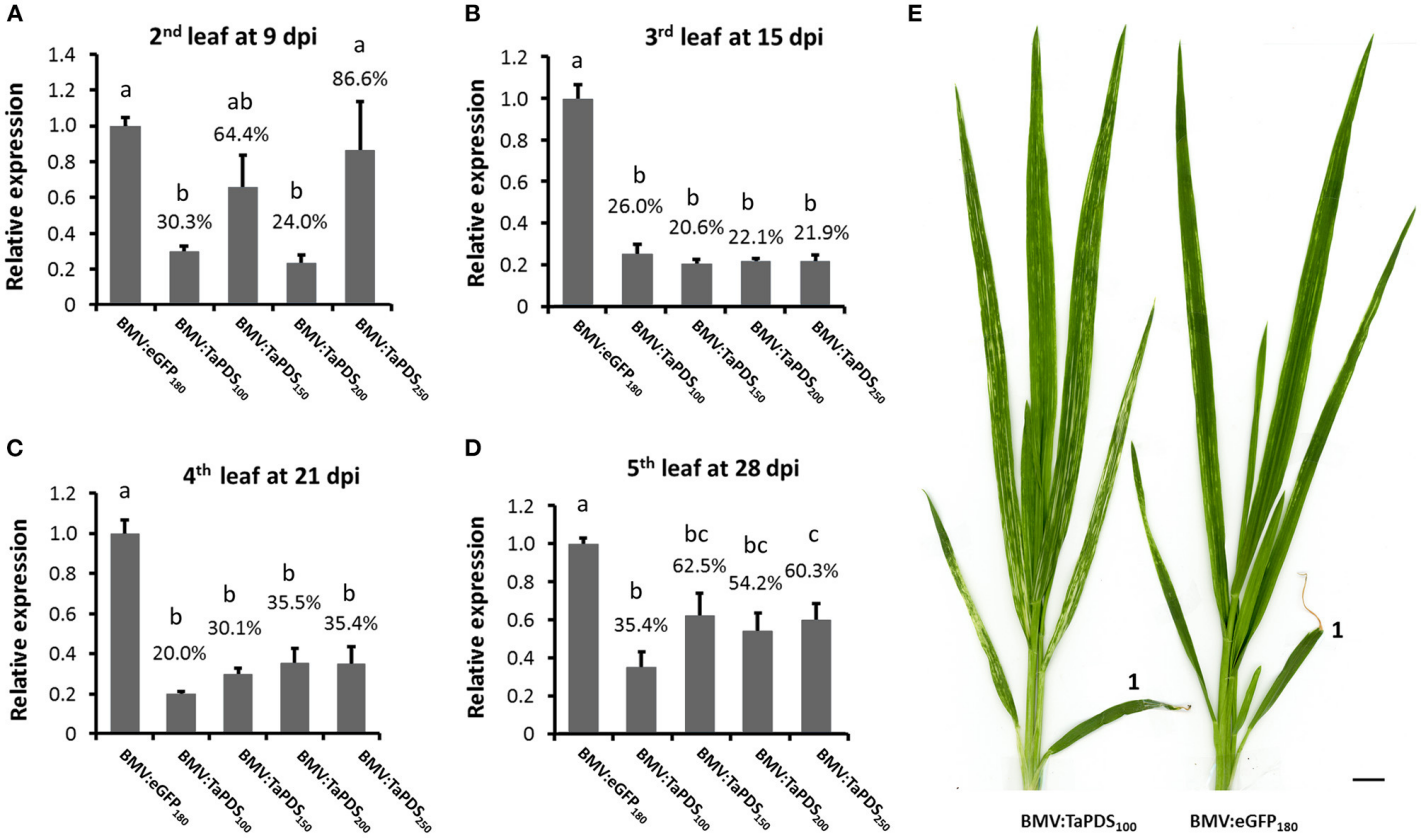
Time-course analysis of BMVCP5-mediated silencing of *TaPDS* in wheat leaves. **(A–D)** Groups of five wheat plants were inoculated with BMVCP5 containing *TaPDS* fragments of 100, 150, 200, or 250 nt, or a 180-nt *eGFP* fragment using the coleoptile inoculation method. Relative expression levels of *TaPDS* mRNA in various systemically-infected leaves at various dpi were determined by RT-qPCR with values across treatments normalized against relative *TaEF1*α mRNA levels. Expression percentages are given relative to the BMV:eGFP_180_ control. Values represent means + SE of five biological replicates for all treatments except BMV:TaPDS_150_, in which four biological replicates were used. Different letters denote significant difference between treatments (*P* < 0.05, significant ANOVA followed by LSD analysis). **(E)** Plants infected by BMV:PDS_100_ or BMV:eGFP_180_ at 28 dpi. The first leaf above the inoculated coleoptile is marked with “1.” Bar = 1 cm. A consistent bleaching phenotype and silencing of *TaPDS* transcripts were detected in the 2nd to 5th systemic leaves of wheat plants infected with BMV:PDS_100_. Similar results were obtained in two independent experiments.

Target gene transcript silencing, along with silencing efficiency and effectiveness, were further investigated in wheat leaves using the coleoptile inoculation method and the BMV vector containing *TaPDS* fragments of various lengths. When coleoptiles were inoculated with BMV:TaPDS_100_ there was >64% decrease in the level of *TaPDS* transcript in the 2nd to 5th systemic leaves relative to the BMV:eGFP_180_ control (Figures 3A–D). Bleaching was observed in the 2nd to 5th systemic leaves infected with BMV:TaPDS_100_ compared with the sparse green-yellow streaking phenotype associated with leaves infected with BMV:eGFP_180_ (Figure 3E). Thus, the coleoptile inoculation method induced efficient and effective visible silencing. For the larger inserts (150, 200, and 250 nt), the level of target transcript silencing varied but generally decreased more rapidly than that induced by the 100-nt insert over time (Figures 3A–D; Supplementary Figure 4A shows transcript values for individual plants from each treatment). Consistent with previous observations, the bleaching phenotype looked more intense (whiter) but the silencing frequency (number of plants showing silencing/total plants treated) and effectiveness were decreased for the longer inserts compared with the 100-nt insert (Table 1). This loss in target gene transcript silencing was positively correlated with the relatively quicker loss of the insert in succeeding leaves from the vectors with inserts longer than 100 nt (Supplementary Figure 4B).

**Table 1:** Comparison of the infection and silencing features of BMVCP5 containing different *TaPDS* inserts.

**VIGS vector**	**Infection frequency (%)^a^**	**Silencing frequency (%)^b^**	**Number of plants with bleached leaf**			
			**L2^c^**	**L3^c^**	**L4^c^**	**L5^c^**
BMV:TaPDS_100_	76.9 (10/13)	100.0 (10/10)	10	10	10	10
BMV:TaPDS_150_	88.9 (8/9)	75.0 (6/8)	4	6	6	0
BMV:TaPDS_200_	72.7 (8/11)	87.5 (7/8)	7	7	5	0
BMV:TaPDS_250_	66.7 (8/12)	87.5 (7/8)	0	5	7	0
BMV:eGFP_180_	91.7 (11/12)	0.0 (0/11)	0	0	0	0

*Coleoptiles of 3 day-old wheat seedlings (cv. Overley) were rub-inoculated with sap from N. benthamiana leaves infiltrated with BMV VIGS vectors. Phenotypic data collected at 28 dpi*.

a *Calculated by dividing the number of plants infected by the total plants inoculated*.

b *Calculated by dividing the number of plants showing bleaching phenotype by the total plants infected*.

c *Systemically infected leaf (L) designated from bottom to top (2nd to 5th leaf above coleoptile)*.

To investigate whether an even shorter insert in BMVCP5 could induce gene silencing, a 52-bp *TaPDS* fragment, which overlapped with the 100-bp insert tested above, was cloned into BMVCP5 for wheat infection. No bleaching phenotype was observed on any systemic leaves from a total of over 20 infected plants in two independent experiments (Supplementary Figure 5A). However, RT-qPCR analysis revealed knockdown of *TaPDS* transcripts to 32% of control tissue values in the 4th systemic leaf at 25 dpi (Supplementary Figure 5B), in which the majority of samples maintained a vector with full-length insert (Supplementary Figure 5C).

### BMVCP5-Induced Gene Silencing in Wheat Roots

**Figure 4:**
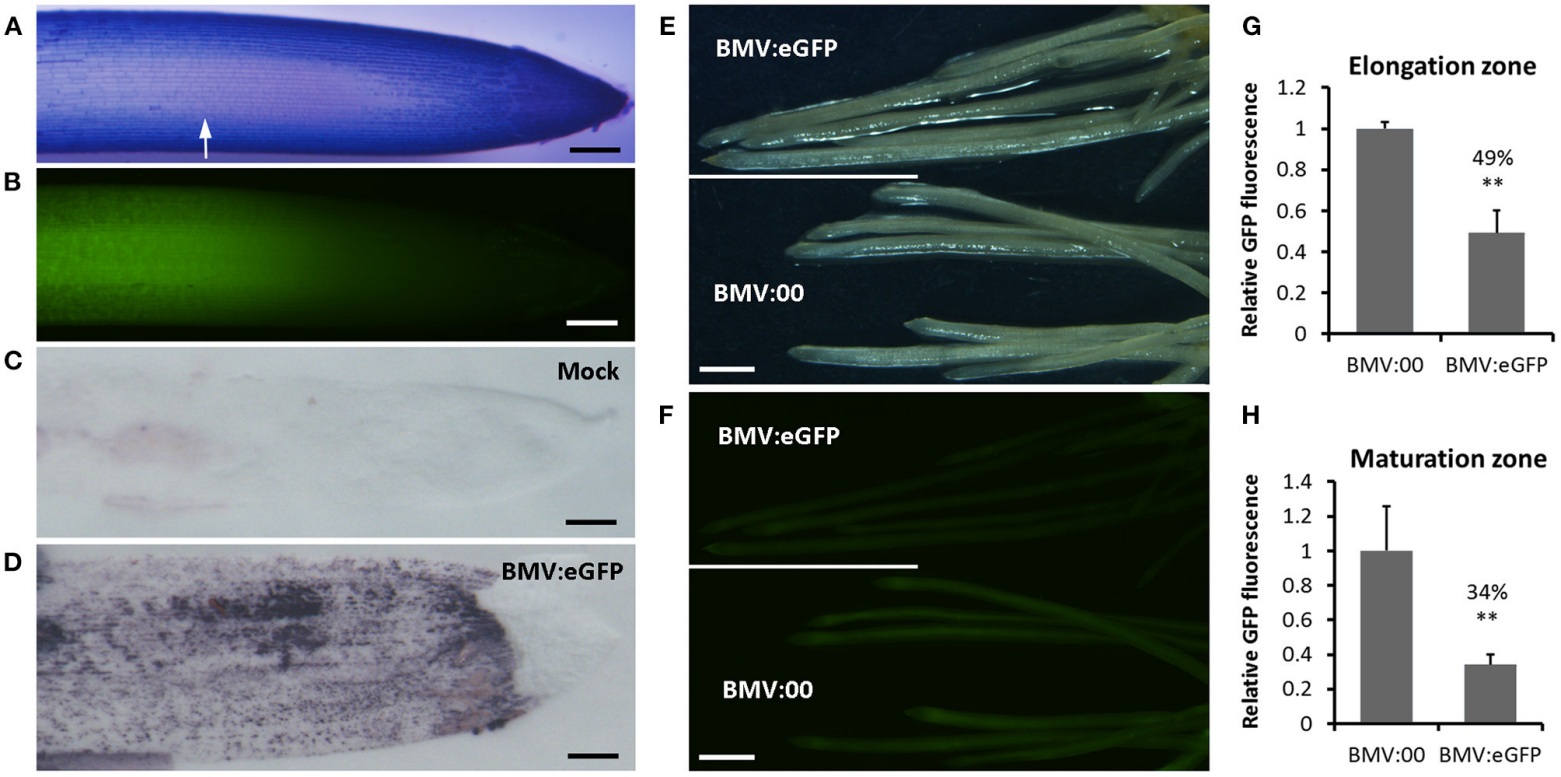
Silencing transgenically-expressed GFP in wheat roots with BMVCP5. **(A)** Root apical region from transgenic OsRCg2GFP wheat (*cv*. fielder) seedlings expressing GFP stained with toluidine blue. White arrow indicates the start of the elongation zone. **(B)** Green fluorescence from GFP detected in the elongation zone of the same root as in panel **(A)**. Accumulation of BMV in a wheat root from plants inoculated with sap of *N. benthamiana* leaves without virus (Mock, **C)**, or containing BMV:eGFP (180-nt insert; **D**), detected by tissue-print assay using a polyclonal antibody against the BMV CP. Purple color indicates presence of virus. Bars in **(A–D)** = 200 μm. **(E)** Bright-field image of roots at 21 dpi from plants infected with BMV:eGFP or BMV:00 (no insert control). **(F)** Fluorescence from roots in panel **E** showing silencing of GFP in transgenic OsRCg2GFP wheat roots after infection with BMV:eGFP, but not BMV:00. Images taken with an Olympus SZX 12 fluorescence microscope. Bars **(E,F)** = 1 mm. Similar results were obtained in three independent experiments. Quantification of GFP fluorescence in the elongation zone **(G)** and maturation zone **(H)** of roots at 21 dpi from plants infected with BMV:00 or BMV:eGFP. Values represent means + SD of six to eight biological replicates, and treatment values compared (*t*-test; **P < 0.01). Percentage values are relative to the BMV:00 treatment.

To explore whether BMVCP5 can mediate gene silencing in roots, VIGS of a visual marker gene, *eGFP*, in homozygous T3 lines of transgenic OsRCg2GFP spring wheat (*cv*. Fielder) was investigated. Concurrently, the accumulation of BMVCP5 in roots of inoculated plant was detected by an antibody-based tissue-blotting assay (Nelson et al., 1993). Without BMVCP5 infection, GFP fluorescence was primarily detected in the elongation zone and the central stele of the mature region of the root (Xue et al., 2016, Figures 4A,B), and no or very weak background signal of the BMV capsid protein was observed after probing tissue with polyclonal antibody against the BMV capsid protein (Figure 4C). Upon infection with BMV:eGFP_180_, strong signal representing BMV capsid protein was exhibited in all root regions except the root cap (Figure 4D), indicating the presence of BMVCP5 and the potential to silence gene expression in those root regions. Relative to roots infected with BMVCP5 without an insert (BMV:00), GFP fluorescence was greatly decreased in both the elongation zone and the mature region of roots at 21 dpi with BMV:eGFP_180_ (Figures 4E,F), with 51 and 66% reduction, respectively, compared to the BMV:00 control (Figures 4G,H), indicating BMVCP5 significantly silenced target gene expression in the host roots.

**Figure 5:**
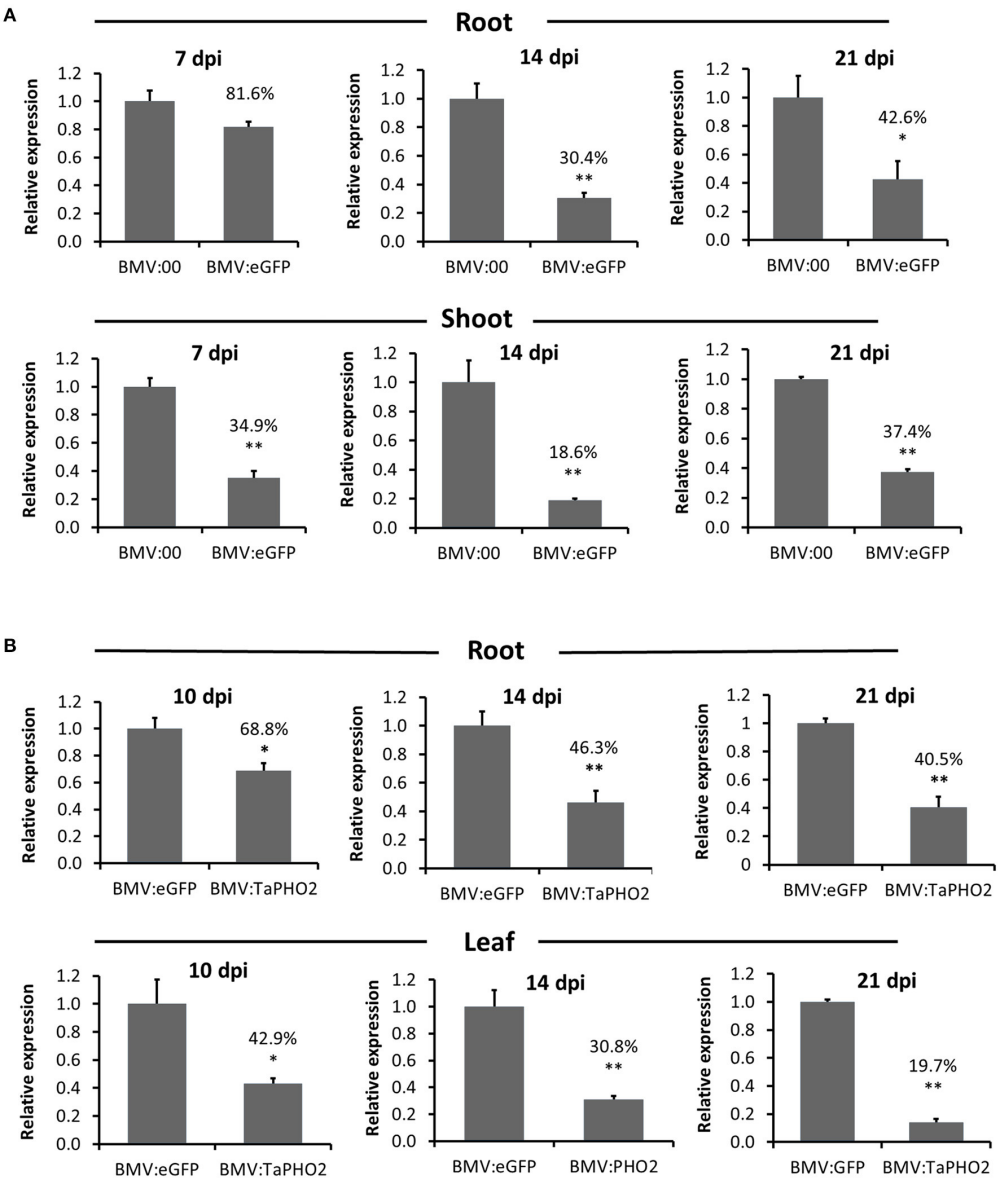
Comparison over time of BMVCP5-mediated silencing of *eGFP* and *TaPHO2* in wheat root and aerial tissues. **(A)** Relative expression levels of *eGFP* mRNA in roots and shoots from transgenic OsRCg2GFP wheat (*cv*. Fielder) plants infected with BMV:eGFP (180-nt insert) or BMV:00 (no insert control) at 7, 14, and 21 dpi. Percentage values are relative to the BMV:00 control. Values represent means + SE of four to seven biological replicates, and treatment values compared (*t*-test: *P < 0.05; **P < 0.01). **(B)** Relative expression levels of *TaPHO2* mRNA in roots and leaves from wheat plants (*cv*. Overley) inoculated with BMV:TaPHO2 (114-nt insert) or BMV:eGFP (107-nt insert) at 10, 14, and 21 dpi. Target gene mRNA levels were analyzed by RT-qPCR, and values normalized against relative *TaEF1*α mRNA levels. Percentage values are relative to the BMV:eGFP (107-nt insert) control. Values represent means + SE from three to six biological replicates, and treatment values compared (*t* test; *P < 0.05; **P < 0.01). Similar results were obtained in two independent experiments.

To further understand the silencing characteristics of BMVCP5 VIGS in wheat roots and compare them with those in aerial tissues, time-course experiments were performed targeting *eGFP* in transgenic OsRCg2GFP spring wheat and *TaPHO2* in winter wheat (*cv*. Overley). After an initial lack of effect at 7 dpi with BMV:eGFP_180_, *eGFP* mRNA in roots was significantly decreased, exhibiting ~37 and 44% of the level in roots infected with BMV:00 at 14 and 21 dpi, respectively (Figure 5A). By contrast, higher percentages of *eGFP* silencing were detected in shoots of plants infected with BMV:eGFP_180_ at 7, 14, and 21 dpi than in the root tissue (Figure 5A). Transcript silencing of *TaPHO2* in root tissue was observed at all dpi with BMV:TaPHO2_114_: ~69, 46, and 41% relative to transcript level present in root tissue infected with BMV:eGFP_107_ at 10, 14, and 21 dpi, respectively (Figure 5B). Interestingly, as observed for *eGFP* silencing in shoots vs. roots, higher percentages of *TaPHO2* mRNA silencing were detected in leaves vs. roots at all time-points (Figure 5B).

To determine whether the decreased silencing in roots also was correlated with more rapid loss of the inserts in the BMVCP5 vector in this tissue vs. the aerial tissue, insert stability was analyzed for the samples described in the previous paragraphs. The 180-nt *eGFP* insert showed similar stability in shoots and roots at each of the three time points (Supplementary Figure 6A). The 114-nt *TaPHO2* insert was more stable in leaf samples than in the root samples at each time point (Supplementary Figure 6B). In a separate experiment analyzing silencing of *TaPDS* after inoculation with BMV:TaPDS_100_, *TaPDS* mRNA expression was silenced to a higher degree in the 5th systemic leaf than in the root at 28 dpi, 86.5% reduction in leaves vs. 56.3% reduction in roots (Supplementary Figure 7A). However, as for *eGFP* silencing, the 100-nt *TaPDS* insert showed similar stability in the 5th systemic leaf and roots (Supplementary Figure 7B). Therefore, the decreased level of transcript silencing in the root by BMVCP5 may be due to other factors than just the stability of the insert in the virus vector in that tissue.

### BMVCP5-Mediated *TaPHO2* Silencing Led to Increased Phosphate Accumulation in Leaves

**Figure 6:**
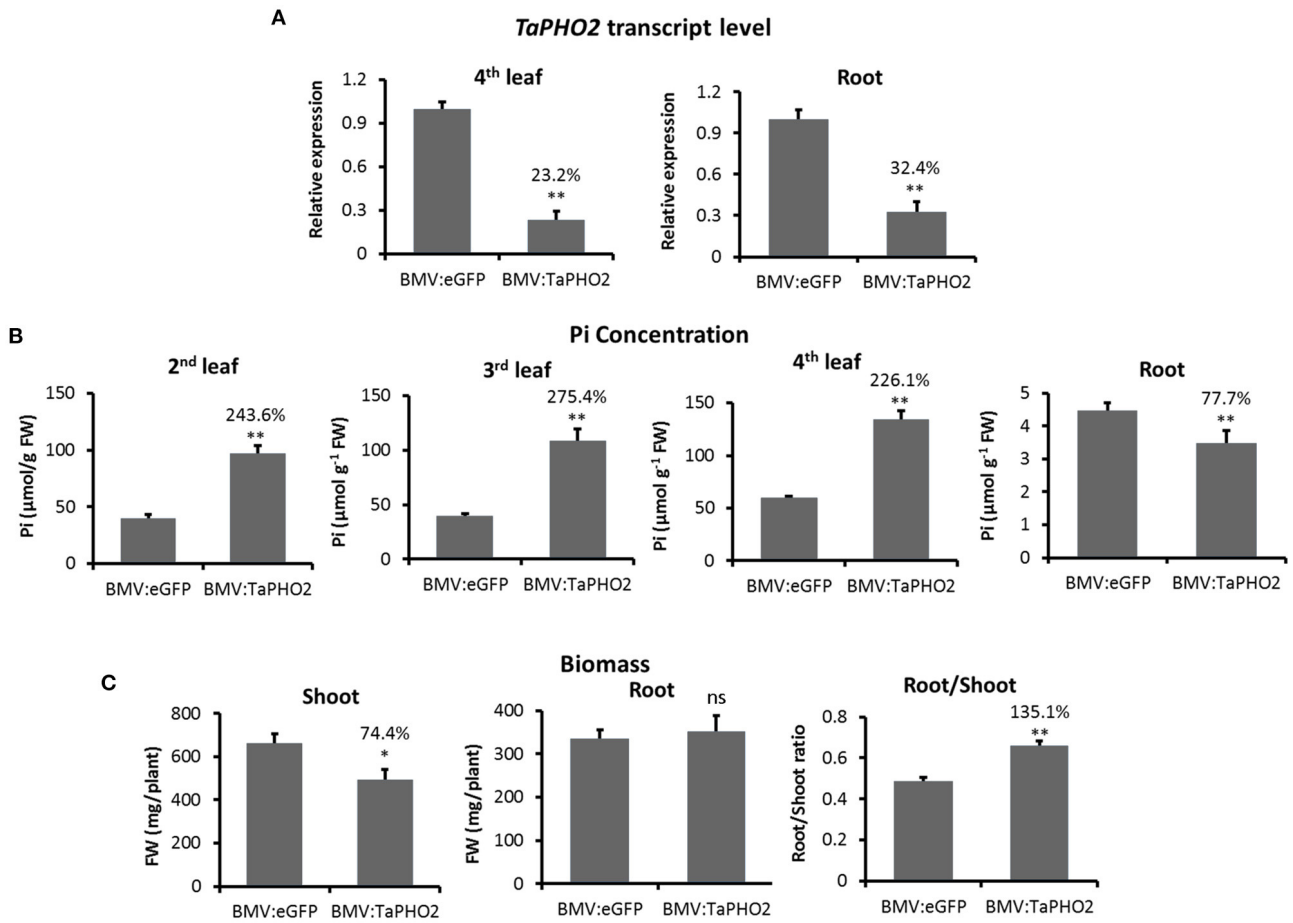
Inorganic phosphate concentration and biomass of wheat plants silenced for *TaPHO2* expression with BMV:TaPHO2_114_ infection. Wheat (*cv*. Overley) plants inoculated with BMV:TaPHO2 (114-nt insert) or BMV:eGFP (107-nt insert; control) were grown under a low phosphate condition (20 μM Pi). Samples were collected at 24 dpi. **(A)** *TaPHO2* expression levels in the 4th systemic leaf or roots, determined by RT-qPCR with values across treatments normalized against relative *TaEF1*α mRNA levels. **(B)** Pi concentrations in the 2nd, 3rd, or 4th systemic leaves or roots of plants inoculated with BMV:TaPHO2 or BMV:eGFP. **(C)** Shoot biomass, root biomass and Root/Shoot ratio of plants inoculated with viruses. Percentage values are relative to the BMV:eGFP control. Values represent means + SE of five biological replicates and treatment values compared (*t*-test; *P < 0.05; **P < 0.01). Similar results were obtained in two independent experiments. ns, not significant.

To determine whether BMV VIGS resulted in a modified biochemical phenotype expected due to down-regulated expression of a target gene, the effect of BMVCP5-mediated *TaPHO2* silencing on Pi accumulation in wheat plants was analyzed. In wheat plants infected with BMV:TaPHO2_114_, *TaPHO2* transcripts were decreased to ~23% in the 4th systemic leaf and 32% in roots at 25 dpi compared with similar tissue infected with BMV:eGFP_107_ (Figure 6A). Pi concentrations in the 2nd, 3rd, and 4th systemic leaves from plants with decreased *TaPHO2* mRNA expression were over twice the level of Pi in tissues infected with BMV:eGFP_107_ (Figure 6B). However, in roots of the *TaPHO2*-silenced plants Pi concentration was reduced by 22% compared with the BMV:eGFP_107_ control (Figure 6B). The *TaPHO2*-silenced plants also had a lower shoot biomass but similar root biomass (Figure 6C), which led to an increased root/shoot ratio in comparison to the control (Figure 6C).

### BMVCP5 as a Potential VIGS Tool in Various Wheat Cultivars

In order to determine the range of wheat varieties to which the BMV VIGS system could be applied, a total of 54 wheat cultivars were screened for susceptibility to virus infection by BMV:GFPuv, a vector containing a fragment from a variant GFP (Ding et al., 2018), using the leaf blade inoculation method. All wheat cultivars were susceptible to BMV:GFPuv infection, displaying diverse disease symptoms in systemically-infected leaves. The disease symptoms from the 54 wheat cultivars were divided into three categories according to the severity: (1) mild (mild light green mottling or streaking; 10 cultivars), (2) moderate (mild or moderate yellow mosaic; 17 cultivars), and (3) severe (yellowing or severe yellow mosaic; 27 cultivars). Plant height reduction was related to the severity of virus symptoms (Supplementary Table 1).

**Figure 7:**
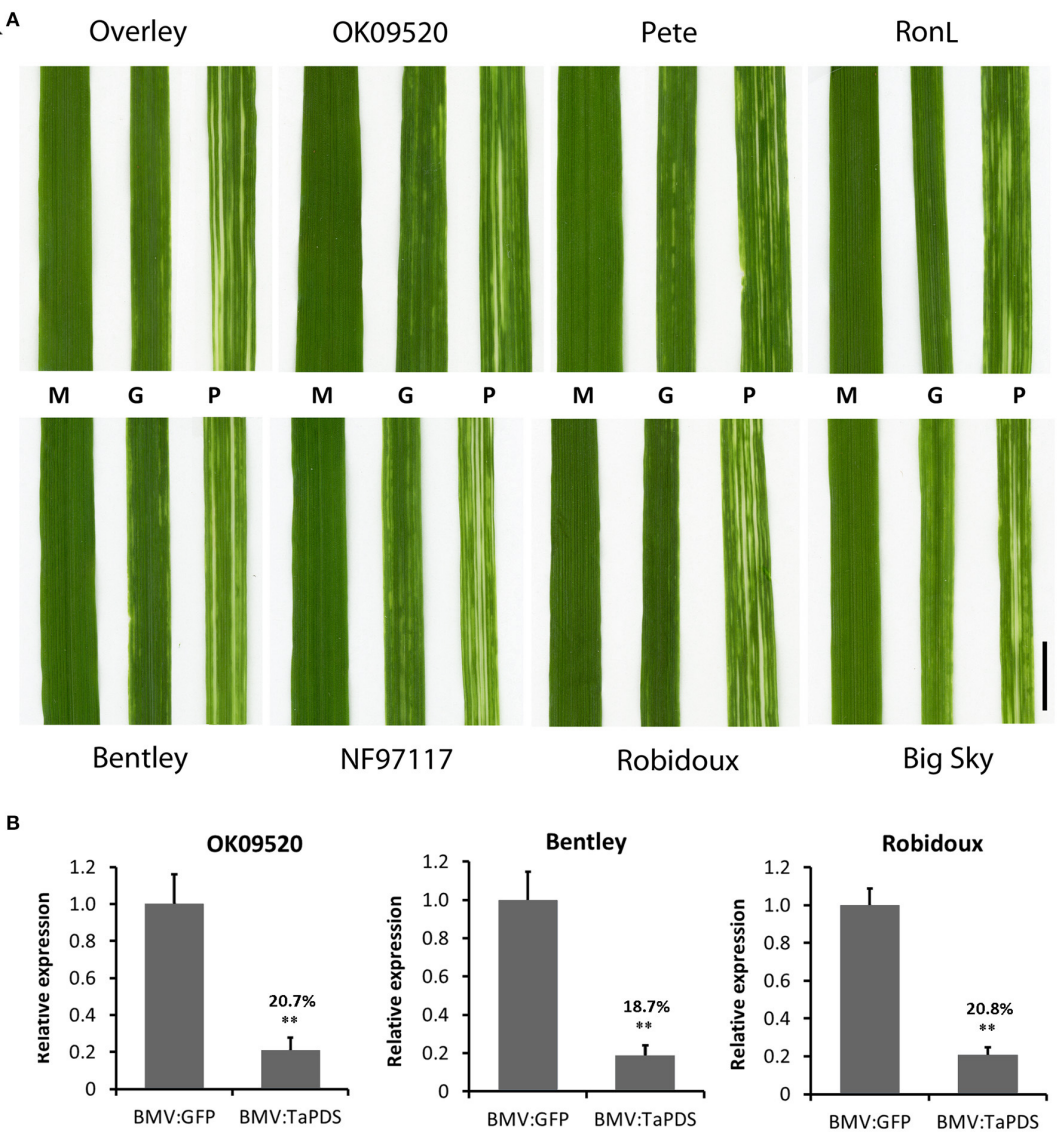
BMVCP5-induced *TaPDS* silencing in eight winter wheat cultivars. **(A)** Images show a representative phenotype of the 3rd systemic leaf at 22 dpi from plants infected with mock (M), BMV:eGFP_107_ (G) or BMV:TaPDS_100_ (P). Bar = 1 cm. **(B)** *TaPDS* mRNA expression levels in the 3rd systemic leaf at 22 dpi from plants infected with BMV:eGFP (107-nt insert) or BMV:TaPDS (100–nt insert) in cultivars OK09520, Bentley and Robidoux, analyzed by RT-qPCR with values across treatments normalized against the transcript level of *TaEF1*α. Expression percentages are given relative to the BMV:eGFP control. Values represent means + SD of five biological replicates, and treatments compared (*t*-test; **P < 0.01). Similar results were obtained in two independent experiments.

Next, the effect of BMVCP5-induced *TaPDS* silencing was analyzed in eight wheat cultivars, including four with mild disease symptoms (Overley, OK09520, Pete, and RonL), three with moderate disease symptoms (Bentley, NF97117, and Robidoux) and one with severe disease symptoms (Big Sky). Seventeen to twenty plants from each cultivar were infected with BMV:eGFP_107_ or BMV:TaPDS_100_ by the leaf inoculation method. In the eight cultivars, infection frequencies ranged from 95 to 100% for BMV:eGFP_107_ and 74 to 100% for BMV:TaPDS_100_. Streaking or mottling symptoms of varying degrees were observed in all cultivars inoculated with BMV:eGFP_107_. The frequency of visible VIGS phenotype induced by BMV:TaPDS_100_ infection, characterized by bleaching in infected leaves (Figure 7A), varied from 74 to 100% among cultivars (Table 2). Bleaching usually developed from the 2nd to 5th systemic leaves in the eight cultivars, with the strongest bleaching occurring on the 3rd and 4th leaves.

**Table 2:** BMV:TaPDS_100_-induced *TaPDS* silencing in eight wheat cultivars.

**Cultivar**	**Infection frequency (%)^a^**	**VIGS frequency (%)^b^**
Overley	100.0 (17/17)	82.4 (14/17)
OK09520	94.7 (18/19)	77.8 (14/18)
Pete	94.7 (18/19)	83.3 (15/18)
RonL	90.0 (18/20)	100.0 (18/18)
Bentley	100.0 (19/19)	73.7 (14/19)
NF97117	78.9 (15/19)	86.7 (13/15)
Robidoux	100.0 (18/18)	77.8 (14/18)
Big Sky	73.7 (14/19)	92.9 (13/14)

*The first leaves of 8 day-old wheat seedlings were rub-inoculated with sap from N. benthamiana leaves infiltrated with BMV VIGS vectors. Phenotypic data collected at 21 dpi*.

a *Calculated by dividing the number of plants infected by the total plants inoculated*.

b *Calculated by dividing the number of plants showing bleaching phenotype by the total plants infected*.

*TaPDS* mRNA silencing was verified in the 3rd systemic leaves of OK09520, Bentley and Rubidoux. An ~80% decrease in *TaPDS* transcript levels was detected in leaf extracts from all the three cultivars compared with the BMV:eGFP controls (Figure 7B). These results demonstrate the efficacy of BMVCP5-mediated VIGS in various wheat genotypes.

## Discussion

### Factors Affecting BMVCP5-Mediated VIGS

VIGS studies have found that insert stability in a VIGS vector is positively correlated with the ability of the VIGS system to silence expression of the target gene (Ramanna et al., 2013). Therefore, improving the stability of foreign inserts in the virus vector during infection appears to be critically important for a VIGS system. Using our silencing protocol for wheat, the BMVCP5 silencing vector was amplified in an intermediate host, *N. benthamiana*, and then extract from the inoculated leaves was inoculated to wheat. Therefore, insert stability in both hosts was important to obtain maximum silencing in wheat. For the three target genes there was no loss of insert at 180 nt or less from the BMVCP5 vector in *N. benthamiana* extract at 4 days post infiltration (Supplementary Figure 2). Full insert stability was observed previously with this vector expressing a maize 250-nt PDS gene fragment in *N. benthamiana* at 3 days post infiltration, with minor loss at 6 days post infiltration (Ding et al., 2018). We previously recommended analyzing insert stability in *N. benthamiana* extracts to select extract with the most intact insert for inoculation to the monocotyledonous host (e.g., Zhu et al., 2014; Ding et al., 2018). Our current findings suggest that when using inserts of optimal length (~100 nt) it is not necessary to check insert stability in the *N. benthamiana* inoculum. This will shorten the time and lessen the associated costs when using BMV-mediated VIGS in all monocotyledonous species where *N. benthamiana* is an intermediate host.

In wheat leaves, small inserts around 100 nt were much more stable than larger inserts (150–252 nt) and resulted in consistent and substantial silencing of the target gene mRNA (Figure 3, Supplementary Figure 4); loss of the larger inserts was correlated with higher target gene mRNA expression. A negative correlation between insert length and insert stability and/or silencing efficiency also was reported for other VIGS systems, such as *Potato virus X-*VIGS in *N. benthamiana* (Avesani et al., 2007), BSMV-VIGS in barley (Bruun-Rasmussen et al., 2007) or *N. benthamiana* (Yuan et al., 2011) and CWMV-VIGS in wheat (Yang et al., 2018). In maize, stability of 250-nt inserts (only size studied) in BMVCP5 was significantly improved over that in the unimproved BMVF13m vector, but partial loss of the full length inserts of *ZmPDS* or *ZmHSP70-1* was apparent even at 5 dpi in inoculated leaves and 5 dpi (*PDS*) or 10 dpi (*HSP70-1*) in systemically-infected leaves (Ding et al., 2018). Additionally, using the original *in vitro* transcription-based vector, C-BMV_A/G_, it was reported that an 86-nt PDS insert induced extensive light-yellow and white streaks in systemic leaves of maize and rice and significant silencing of the target transcript (Ding et al., 2006), suggesting stability for this small insert in the unimproved vector. These studies indicate that an insert of ~100 nt is the optimal size for the BMVCP5 vector in wheat and likely for other host species.

Interestingly, infection with BVMCP5 containing an even smaller insert, 52-nt *TaPDS*, triggered no bleaching phenotype (Supplementary Figure 5), but knocked down target mRNA by 68%, a decrease comparable to that induced by larger inserts (compare Figure 3D with Supplementary Figure 5B). Similar findings were reported in a BSMV-VIGS study in barley, where a 128-nt *PDS* fragment caused a significant reduction in *PDS* mRNA levels but no bleaching (Bruun-Rasmussen et al., 2007). The cause of disconnect between target mRNA downregulation and visible symptoms for these smaller inserts is not understood. Bruun-Rasmussen et al. (2007) speculated that to trigger a visible bleaching phenotype *PDS* mRNA levels should be lower than a specific threshold at a certain point in leaf development. Additionally, it is possible that mRNA silencing may be present for a longer period of time, but at a consistently lower level, in more leaf cells from plants infected with BMV:TaPDS_52_ than with BMVCP5 expressing larger *TaPDS* inserts. The instability of larger *TaPDS* inserts would lead to little or no silencing in more leaf cells than during BMV:PDS_52_ infection and therefore an artificial similarity in measured silencing degrees per whole leaf between BMV:PDS_52_ and BMV with larger *TaPDS* inserts. More precise research analyzing different portions of leaf showing patches of visible silencing for presence of insert and level of silencing is necessary to clarify whether both, one or neither interpretation has merit.

Besides insert size, insert sequence may also affect insert stability. In our study, the 180-nt *eGFP* insert was more stable than the 150-nt *TaPDS* or *TaPHO2* insert (Figure 2). Ding et al. (2006) found a 398-nt insert from an actin gene was more stable than a 240-nt insert from a *PDS* gene in maize with the BMV vector. In a BSMV-VIGS study in barley roots, Pacak et al. (2010b) reported similar findings: a 251-nt insert from the barley *IPS1* gene and a 387-nt insert from the barley *PHO2* gene were much more stable than a 250-nt *GFP* insert. Studies showed that both the viral genome length and structure have been optimized during evolution to enhance the formation of stable viral particles (Perlmutter et al., 2013). For VIGS studies, increases in insert length or insertion of sequence that affects the secondary and tertiary structure of the viral RNA may lead to poor encapsidation, selective encapsidation (insert loss) and native virus spreading. Bruun-Rasmussen et al. (2007) observed that BSMV RNA that had lost insert accumulated to higher levels than BSMV RNA with insert. Additional to encapsidation, the ability of the specific genomic viral RNA with insert and its encoded proteins to accumulate and the ability of the genomic viral RNA with insert to recombine should be considered in future research to fully understand selection exerted by insert sequence or size.

It was interesting that the three *TaPHO2* homoeologs were silenced to different degrees by BMVCP5 containing the 114-nt *TaPHO2* fragment (Figure 2F), which possessed 100% identity with each homoeolog in the targeted region (Supplementary Figure 1B). This result suggests that the target mRNA secondary structure may affect its interaction with the silencing machinery and therefore its subsequent silencing. Evidence from siRNA-based studies showing target mRNA structure affects siRNA silencing efficiency supports this hypothesis (Shao et al., 2007; Gredell et al., 2008). VIGS is a technology based on the plant siRNA-mediated antiviral defense mechanism, therefore factors affecting siRNA silencing efficiency may also influence VIGS silencing efficiency. Selectively silencing particular homoeologs during VIGS through structure-based differential target gene susceptibility is worthy of further study. The siRNA-Finder (si-Fi) software, si-Fi21, offers effective long RNA interference sequence design (Lück et al., 2019). Future VIGS study may consider using this software for selection of insert sequences.

Many VIGS studies report that inoculation methods affect the success of virus vector delivery and the silencing efficiency. Broderick and Jones (2014) compared four inoculation methods when optimizing the *tobacco rattle virus*-based VIGS protocol in petunia (*Petunia* × *hybrida*), and found inoculation of mechanically wounded shoot apical meristems induced the most effective and consistent silencing. Besides the inoculation method, the plant developmental stage may also affect silencing. A TRV-VIGS study in *Antirrhinum* showed that inoculation of plants at first- and second-leaf stages were more effective at inducing silencing than at fifth-leaf stage (Tan et al., 2020). Several groups reported successful delivery of BMV, BSMV and a *cucumber mosaic virus*-based VIGS vector in maize by vascular puncture inoculation of maize seeds (Benavente et al., 2012; Wang et al., 2016; Jarugula et al., 2018). However, direct inoculation of wheat seeds via the vascular puncture method was difficult and the survival rate of the inoculated seeds was low due to the small size of the wheat embryo (Y Wang and RS Nelson, unpublished data). The coleoptile inoculation method developed in this study gave a similar inoculation efficiency and silencing efficiency to that of leaf blade inoculation but allowed inoculation at an earlier seedling developmental stage (3 days post germination). Other inoculation methods, such as seed imbibition or biolistic inoculation (Cheuk and Houde, 2017; Mei et al., 2019), should be investigated for use with the BMVCP5 vector.

We found BMVCP5-VIGS efficiency varied in tissues. Compared with leaf or shoot tissue, BMVCP5 exhibited a relatively lower silencing ability in roots, as revealed by parallel analysis of aerial and root tissue from plants silenced for *TaPDS, TaPHO2* or ectopic *eGFP* mRNA expression (Figure 5, Supplementary Figure 7). For the BSMV vector, Pacak et al. (2010b) reported that instability of inserts may be a more severe problem for silencing of genes in roots compared to leaves and advised screening virus constructs for stability. In our studies a difference in insert stability did not correlate with the generally less efficient silencing in root vs. aerial tissue for these inserts (Figure 5, Supplementary Figures 6, 7). It is possible that the lower silencing ability of BMVCP5 in roots may be associated with differences between root and aerial tissue (e.g., their subcellular constitution) that inhibits accumulation in roots.

### BMVCP5 Is a Valuable Tool for Functional Genomics Studies in Wheat

We demonstrate that BMVCP5 can be used for rapid gene function characterization in young wheat seedlings. Over 50% reduction of the target mRNA was detected in shoots, leaves and roots within 2–4 weeks after inoculation (Figures 2, 3, 5, Supplementary Figures 3, 7), which is good for various phenotyping experiments. By skipping the insert stability analysis in *N. benthamiana*, this new BMVCP5 VIGS procedure is easier to use and more time- and cost-efficient. In addition, using a small insert (~100 nt) for silencing in BMVCP5 is particularly useful for gene function characterization in hexaploid wheat because it is relatively easy to find a short fragment with high specificity targeting a single or multiple genes.

Many root genes, especially those responsive to abiotic stresses or associated with nutrient use efficiency, influence the sustainability of plant production under adverse conditions. Characterization of root genes contributing to, and breeding for adapted root system architecture with improved nutrient and water acquisition or use efficiency will address challenges for modern agriculture such as increasing food security and decreasing environmental impact (Comas et al., 2013; Lynch, 2019). Few studies have analyzed VIGS activity in and its effect on roots of monocotyledonous species (Pacak et al., 2010b; Bennypaul et al., 2012; He et al., 2015). In our study, BMVCP5-induced *TaPHO2* silencing led to Pi accumulation in wheat leaves (Figure 6B), consistent with previous findings using *PHO2* mutants in Arabidopsis (Aung et al., 2006; Bari et al., 2006), or wheat (Ouyang et al., 2016), and BSMV VIGS of *HvPHO2* in barley (Pacak et al., 2010b). As in our study (Figure 6B), the latter two studies reported decreased root Pi concentrations when knocking out any *TaPHO2* homoeolog in wheat or *HvPHO2* in barley. These results indicate that silencing *TaPHO2* expression via BMVCP5 functionally mimicked findings with stable mutants of *PHO2* in various plant species, underlining the value of BMVCP5 for functional genomic studies in wheat.

Genetic resources and the genetic diversity embodied by those resources are fundamental for sustaining wheat production and critical for enhancing yield potential (Tadesse et al., 2016). Intensive efforts have been made to collect and conserve wheat genetic resources and attain genome-wide genetic variation (Jordan et al., 2015; Tadesse et al., 2016; Liu et al., 2017; Zhou et al., 2017). To take advantage of the wide range of genetic diversity from these abundant genetic resources, gene functional characterization in diverse breeding lines or cultivars is indispensable. Of the few functional genomics tools in wheat, stable transformation and mutant collections are limited to only a few genotypes (Chen et al., 2012; Dhaliwal et al., 2015; Fitzgerald et al., 2015; Guo et al., 2017; Hayta et al., 2019; Wang et al., 2020), while VIGS allows for rapid gene function characterization in many wheat cultivars or breeding lines, as shown by many BSMV VIGS studies (referenced in Dommes et al., 2019). In this study, we demonstrated BMVCP5 had a high infection capacity in wheat (100% infection rate of the 54 wheat cultivars), and half of the screened cultivars exhibited mild or moderate disease symptoms. Moreover, BMVCP5 showed a high success rate of VIGS (100% of plants silenced for *TaPDS* expression displayed a visual bleaching phenotype in all eight cultivars examined (Figure 7A). These results demonstrate the great potential for application of BMVCP5-mediated VIGS in various wheat genotypes. This could be particular useful in studying cultivar specific traits, such as validation of top candidate genes from genome-wide association studies.

Although all VIGS vectors share some common features such as rapid and capable targeting of single or multiple genes, each VIGS system has unique requirements for its best use. These include optimizing insert length, identifying tissue and cultivar susceptibility, optimizing plant growth conditions, and fulfilling biosafety regulations. For BSMV-based VIGS, insert sizes from 250 to 400 bp were more effective over time than one over 500 bp in silencing *MAGNESIUM CHELATASE* (*TaChlH*) expression in wheat (Yuan et al., 2011). CWMV containing a *PDS* fragment of 500 bp provided better silencing than those of 800, 1,000, or 1,500 bp (Yang et al., 2018). Using inverted-repeat fragments of 60 nucleotides from *PDS* or *CLOROPLASTOS ALTERASDOS1* (*CLA1*), FoMV efficiently down-regulated target gene expression in wheat (Liu et al., 2016). Shorter sequences make it easier to avoid off-target silencing. BSMV and BMV vectors silence in multiple tissues of wheat (Figures 2–6; Bennypaul et al., 2012), while the CWMV- and FoMV-VIGS have only been studied in leaves (Liu et al., 2016; Yang et al., 2018). BMV and BSMV function in multiple wheat cultivars (Figure 7; Dommes et al., 2019); while CWMV- and FoMV-VIGS have been investigated in one wheat cultivar (Liu et al., 2016; Yang et al., 2018). CWMV-VIGS in wheat functions well at 17°C (Yang et al., 2018), while BSMV showed good silencing at 18–22°C in wheat (Cakir and Tör, 2010; Bennypaul et al., 2012). BMV- and FoMV-based silencing studies in wheat were conducted at 22/19°C (day/night) and 22–24°C, respectively (this study; Liu et al., 2016). Regarding safety and security issues, BMV is poorly seed transmitted, while BSMV could be transmitted through seed (Mise and Pocsai, 2004; Bruun-Rasmussen et al., 2007). Due to the potential impact of these viruses on agriculture, VIGS vectors are strictly controlled by government safety regulations. BMVCP5-VIGS system provides an additional choice for wheat researchers who (1) are limited by local government restrictions from using other vectors, (2) are concerned about seed transmission, or (3) have experience with BMV and prefer to use it in their functional genomics studies. The addition of BMVCP5 to the list of VIGS tools will further accelerate functional genomics studies and productive breeding in this species.

## Materials and Methods

### Plant Materials and Growth Conditions

All 54 wheat cultivars used in this study are hexaploid. Seeds of 48 winter wheat cultivars were obtained from the Small Grains Laboratory at Noble Research Institute, LLC (Ardmore, USA), and seeds of six cultivars (Eltan, Kavkaz, Apogee, WinTex, Chancellor, Golden Chief) were requested from USDA (Supplementary Table 1). Cultivar Overley was used for cloning *TaPDS* and *TaPHO2* fragments and silencing experiments targeting *TaPDS* and *TaPHO2* expression. Homozygous T3 lines of transgenic *OsRCg2GFP* wheat (background: spring wheat *cv*. Fielder) were generated from the T2 lines (Xue et al., 2016), and used in silencing experiments targeting *eGFP* expression. *N. benthamiana* and wheat plants were grown in the Metro-Mix® 360 medium (Sun Gro Horticulture, USA) in a greenhouse under 16 h light (300–500 μmol m^−2^ s^−1^) at 22°C and 8 h dark at 19°C with 60% relative humidity. For silencing studies targeting *TaPHO2* expression, wheat plants were grown in a mixture of turface:sand:perlite (2:2:1) under low phosphate treatment (20 μM KH_2_PO_4_). For silencing studies targeting *eGFP*, a hydroponic culture system with a half Hoagland nutrient solution (Hoagland and Arnon, 1950) was used under the same greenhouse conditions.

### Construction of BMV VIGS Vectors

PCR primers for cloning of *TaPHO2* and *TaPDS* fragments from wheat (*cv*. Overley) were designed at the conserved regions of the three homoeologs for each gene. The 52-, 100-, 150-, 200-, and 250-bp fragments of *TaPDS* (GenBank accession: FJ517553.1) were amplified by RT-PCR using primer pairs of P1-F4 /P1-R2, P1-F4/P1-R1, P1-F3/P1-R1, P1-F2/P1-R1, and P1-F1/P1-R1, respectively. The 114-, 150-, 204-, and 252-bp fragments of *TaPHO2* (GenBank accession: AK331438.1) were amplified using primer pairs of P2-F1/P2-R4, P2-F1/P2-R3, P2-F1/P2-R2, and P2-F1/P2-R1, respectively. Similarly, The 107-, 180-, and 220-bp *eGFP* (GenBank accession: U55761.1) fragments were amplified from the T3 transgenic *OsRCg2GFP* wheat seedlings using primer pairs P3-F3/P3-R1, P3-F2/P3-R1, and P3-F1/P3-R1, respectively. All forward primers and reverse primers contained, respectively, an *Avr*II (CCTAGG) or *Nco*I (CCATGG) restriction enzyme site. The resulting PCR fragments were cloned into the *Avr*II and *Nco*I sites of the pC13/F3CP5, encoding RNA3 of the BMVCP5 vector. The pC13/F3CP5 plasmid with pC13/F1+2, encoding RNAs 1 and 2 of BMV, composed the BMVCP5 vector and were used in the VIGS experiments. BMV:GFPuv, a BMVCP5 vector with a 250-bp fragment from a variant of the GFP gene (Ding et al., 2018), was used to screen susceptibility of wheat cultivars for BMV infection. Primers used for construction of BMVCP5 VIGS vectors in this study are listed in Supplementary Table 2.

### Total RNA Extraction, RT-PCR, and Real-Time PCR

Plant total RNA was extracted using TRIzol Reagent (Life Technologies, USA) and treated using the TURBO DNA-free™ Kit (Invitrogen, USA) to eliminate genomic DNA contamination before reverse transcription. First-strand cDNA was synthesized from 0.5 μg total RNA by M-MLV reverse transcriptase (ThermoFisher Scientific, USA) using a mixture of oligo (dT_18_) and random primers. Cloning of BMV-RNA3 insert fragments and analysis of BMV insert stability were performed by PCR using Phusion® High-Fidelity DNA Polymerase (NEB, USA) and Taq DNA Polymerase (NEB, USA), respectively, following the manufacturer's instructions. Primer pair P4-F/P4-R flanking the foreign gene insert site in pC13/F3CP5 was used to determine insert stability in both *N. benthamiana* and wheat through RT-PCR analysis. Gene expression analyses were evaluated by real-time PCR (qPCR) using gene-specific primers (not amplifying the insert) and the Power SYBR Green PCR Master Mix kit (Applied Biosystems, USA) on an ABI PRISM 7900 HT Sequence Detection System (Applied Biosystems, USA). The wheat translation elongation factor subunit *EF1*α was used as an internal control for gene expression analyses (Paolacci et al., 2009). All primers used for RT-PCR or qPCR in this study were listed in Supplementary Table 3.

### Agroinfiltration of *N. benthamiana* and Viral Inoculation of Wheat

BMV VIGS vectors were transformed into the *Agrobacterium tumefaciens* strain C58C1 by using the freeze-thaw transformation method (Chen et al., 1994). Agrobacterium was grown in YEP liquid medium containing rifampicin (10 mg/L) and kanamycin (50 mg/L) at 28°C to OD_600_ = 1. *Agrobacterium* cultures containing pC13/F1+2 and pC13/F3CP5 with foreign gene inserts were pelleted, re-suspended in infiltration buffer (10 mM MgCl_2_, 10 mM MES, 0.2 mM acetosyringone, pH 5.5) at equal amounts to a final OD_600_ = 2, and infiltrated into the fully expanded leaves of 3-week old *N. benthamiana* plants. Infiltrated leaves were harvested at 4 days post infiltration, frozen in liquid nitrogen, and stored at −80°C until use. Sap inoculums were prepared by grinding 1 g of infected *N. benthamiana* leaf in 1 ml of 0.1 M potassium phosphate buffer (pH 7) containing 0.15 g carborundum, and then rub-inoculated onto the abaxial surface of the 1st or the 1st and 2nd leaf blade(s) of 7–9 day-old wheat seedlings. Plants after infiltration or inoculation were covered with a clear plastic dome overnight at room temperature, and then grown under greenhouse conditions as mentioned above.

For rub-inoculation of coleoptile, wheat seeds were pre-soaked with sterile water for 3 h, then incubated on wet germination paper at room temperature in the dark for 3 days. Coleoptiles were inoculated in a petri dish plate in the following manner. *N. benthamiana* leaf sap inoculum (0.5 ml) was pipetted to the center of the plate. Then a coleoptile (~2.5 mm in length) was lain flat in the leaf sap and a layer of carborundum dusted on it. Holding the crown region with the thumb and pointer fingers from one hand, the coleoptile was rubbed back and forth parallel to its veins and pressing against the plate several times with the pointer finger from the other hand. The coleoptile was then flipped over and the rubbing step repeated. After inoculation, the wheat seedlings were placed onto the wet germination paper in a new plate, covered with the lid of the petri dish plate and cultured at room temperature overnight before growing them in solid media or hydroponic culture system under greenhouse conditions as mentioned above.

All VIGS experiments were repeated two to three times with 9–19 plants inoculated in each experiment. For insert stability and expression analyses, usually five plants were sampled. Detailed sampling information for each experiments are described in the figure legends.

### Tissue-Printing Assay of BMV Viral Accumulation in Wheat Roots

An antibody-based tissue-blotting assay (Nelson et al., 1993; Ding et al., 2018) was used to analyze BMVCP5 accumulation in wheat roots. Roots of transgenic OsRCg2GFP wheat plants inoculated with BMV:GFP_180_ or no virus (mock-inoculated control), were harvested at 21 dpi. Root prints were made on nitrocellulose membranes (Bio-Rad, USA), and BMV accumulation on membranes was visualized by blotting against a BMV CP-specific antibody as previously described (Ding et al., 1999). Images of GFP fluorescence and BMV accumulation in wheat roots were captured with an Olympus SZX 12 microscope. GFP fluorescence was quantified using the ImageJ software (Schneider et al., 2012).

### Phosphate Concentration Measurement

Pi concentrations were determined using a colorimetric micromethod with modifications (Itaya and Ui, 1966; Pant et al., 2008). Whole root and single leaf tissues from BMVCP5-infected wheat plants at 24 dpi were harvested and homogenized with liquid nitrogen. Sterile deionized water was added to the tissue powder in a ratio of 10 μl water per mg fresh weight. Supernatants containing extracted soluble Pi were collected by centrifuging at 10,000 g for 10 min, and diluted with deionized sterile water, 10 times for root samples and 50 times for leaf samples. The diluted supernatant (10 μl) was mixed with 1 N HCl (100 μl) and a malachite green solution (100 μl; one volume 4.2% (w/v) (NH_4_)_6_Mo_7_O_24_·H_2_O in 5 N HCL and three volumes of 0.2% malachite green dye in water), and incubated at room temperature for 10 min before measuring the absorbance at 630 nm using a TECAN Infinite M200 PRO instrument.

### Statistics

Student's *t*-test or one way ANOVA followed by LSD tests were conducted in Microsoft Excel to determine the significant differences between treatment means.

## Data Availability Statement

The original contributions presented in the study are included in the article/Supplementary Material, further inquiries can be directed to the corresponding author/s.

## Author Contributions

RSN, MCS, and W-RS conceived the project. YW and RSN designed the experiments. CC performed real-time PCR analysis. BK screened wheat cultivars for sensitivity to BMV infection and was involved in planning the *eGFP* VIGS experiment. W-RS helped plan and review the *TaPHO2* studies and streamlined the high-throughput Pi assay. YK did the statistical analysis. YW did all other experiments, analyzed data, and drafted the manuscript. RSN, YK, W-RS, MKU, MCS, and CC critically revised the manuscript. All authors read and approved the manuscript.

## Conflict of Interest

The authors declare that the research was conducted in the absence of any commercial or financial relationships that could be construed as a potential conflict of interest.
