# Supplementary material for: An Efficient Brome mosaic virus-Based Gene Silencing Protocol for Hexaploid Wheat (Triticum aestivum L.)
Source: Front Plant Sci. 2021 Jun 18;12:685187. doi: 10.3389/fpls.2021.685187 (PMC8253535; doi:10.3389/fpls.2021.685187)
Supplement: Supplementary file 1 [file Data_Sheet_1.docx]

Supplementary Material

# Supplementary Figures and Tables

**1.1 Supplementary Figures**

**Figure S1 Sequence alignment of the *TaPDS* (A) and *TaPHO2* (B) inserts and their targets.** Sequences of *TaPDS-A* (Traes_4AS_357A85716.1), *TaPDS-B* (Traes_4BL_20E1038D7.1), *TaPDS-D* (Traes_4DL_B8215DE3A.1), *TaPHO2-A1* (Traes_1AL_E5224B6EA.2), *TaPHO2-B1* (Traes_1BL_241A3B9EF.2), and *TaPHO2-D1* (Traes_1DL_F122253A3.2) were obtained from Phytozome (<https://phytozome.jgi.doe.gov/pz/portal.html>). Primer pairs P1-F1/P1-R1, P1-F2/P1-R1, P1-F3/P1-R1, P1-F4/P1-R1 and P1-F4/P1-R2 were used to clone the 250-, 200-, 150-, 100- and 52-bp *TaPDS* insert fragments, respectively; primer pairs P2-F1/P2-R1, P2-F1/P2-R2, P2-F1/P2-R3, P2-F1/P2-R4 were used to clone the 252-, 204-, 150- and 114-bp *TaPHO2* insert fragments, respectively. Binding sites of the primers used to clone the insert fragments are indicated by arrows. Sequence alignments were analyzed by the online tool of Multiple Sequence Alignments on the BAR (http://bar.utoronto.ca/ntools/cgi-bin/ntools_multiplealign_w_mview.cgi).

**Figure S2 Stability of insert fragments in *N. benthamiana* leaves infected with BMVCP5.** *N. benthamiana* leaves were harvested at 4 days post infiltration with *Agrobacterium* that contained plasmids expressing the BMVCP5 derivatives inserted with fragments of varying size from *eGFP*, *TaPDS* and *TaPHO2*, followed by total RNA extraction and RT-PCR using primers flanking the cloning site (P4-F/P4-R). Insert fragment sizes are shown by numerical subscripts after target gene designation. Each lane represents the PCR product from an individual plant. Similar results were obtained in two independent experiments. The 1 kb plus DNA ladder (M) and a 427-bp PCR product amplified from plasmid pC13/F3CP5 with no insert (EV) were used as size markers.

**Figure S3** **BMVCP5 induced bleaching phenotype and *TaPDS* transcript silencing in the 4^th^ and 5^th^ systemic leaves.** (**A**) Wheat plants infected with BMV:TaPDS_100_ or BMV:eGFP_180_ were photographed at 35 dpi. (**B**) *TaPDS* mRNA expression levels in the 4^th^ systemic leaf at 28 dpi and 5^th^ systemic leaf at 35 dpi from plants infected with BMV:TaPDS_100_ or BMV:eGFP_180_, determined by RT-qPCR with values across treatments normalized against relative *TaEF1α* mRNA levels. Expression percentages are given relative to the BMV:eGFP_180_ control. Values represent means + SE of five biological replicates and treatment values compared (t test; **, P < 0.01). (**C**) RT-PCR analysis of insert stability in BMV:TaPDS_100_ or BMV:eGFP_180_ in the 4^th^ systemic leaf at 28 dpi and 5^th^ systemic leaf at 35 dpi using primers flanking the cloning site (P4-F/P4-R). Each lane represents PCR product from an individual plant. Similar results were obtained in two independent experiments. A 1 kb plus DNA ladder (M) and a 427-bp PCR product amplified from the empty vector of pC13/F3CP5 (EV) were used as size markers.

**Figure S4 Stability of *TaPDS* inserts in BMVCP5 and *TaPDS* silencing in wheat leaves during infection.** Groups of five wheat plants were infected by BMVCP5 derivatives harboring a *TaPDS* fragment (100 – 250 nt) or a 180-nt *eGFP* fragment. (**A**) Relative expression levels of *TaPDS* mRNA in the 2^nd^ to 5^th^ systemic leaves (9 dpi to 28 dpi), analyzed by RT-qPCR (the same data as shown in **FIGURE 3A-D**, here showing results from individual plants), normalized against relative *TaEF1α* mRNA levels. Values represent means of three technical replicates. (**B**) Stability of inserted fragments in the same leaves as in panel **A** was analyzed by PCR using the cDNA prepared for qRT-PCR in **FIGURE 3A-D** and a pair of primers flanking the cloning site (P4-F/P4-R). Each lane represents PCR product from an individual plant. Similar results were obtained in two independent experiments. M, 1 kb plus DNA ladder; EV, a 427-bp PCR product amplified from the empty vector of pC13/F3CP5.

**Figure S5** **Silencing *TaPDS* mRNA in wheat by BMV:TaPDS_52._** (**A**) The 3^rd^ systemic leaves at 25 dpi from plants infected with BMV:eGFP (107-nt insert) or BMV:TaPDS_52_. (**B**) *TaPDS* mRNA levels in the 4^th^ systemic leaf at 25 dpi from plants infected with BMV:eGFP or BMV:TaPDS_52_, analyzed by RT-qPCR, normalized against relative *TaEF1α* mRNA levels. Percentage values are relative to the BMV:eGFP control. Values represent means + SD of six biological replicates, and treatment values compared (t test; **, P < 0.01). Similar results were obtained in two independent experiments. (**C**) Stability of the 52-nt *TaPDS* insert in the 4^th^ systemic leaf at 25 dpi, analyzed by PCR using the cDNA prepared for RT-qPCR in panel **B** and primers flanking the cloning site (P4-F/P4-R). 1-6, PCR product from individual plants; M, 1 kb plus DNA ladder; EV, a 427-bp PCR product amplified from the empty vector of pC13/F3CP5.

**Figure S6 Stability of the *eGFP* or *TaPHO2* inserts in shoot or leaf, respectively, and root during infection**. (**A**) Stability of the 180-nt *eGFP* insert in shoots and roots from plants infected with BMV:eGFP_180_ at 7, 14, and 21 dpi, analyzed by PCR using the cDNA prepared for RT-qPCR in **FIGURE 5A** and primers flanking the cloning site (P4-F/P4-R). (**B**) Stability of the 114-nt *TaPHO2* insert in the 2^nd^ systemic leaf and roots at 10 dpi, the 3^rd^ systemic leaf and roots at 14 dpi, and the 4^th^ systemic leaf and roots at 21 dpi, from plants infected with BMV:TaPHO2_114_, analyzed by PCR using the cDNA prepared for RT-qPCR in **FIGURE 5B** and primers flanking the cloning site (P4-F/P4-R). M, 1 kb plus DNA ladder; EV, a 427-bp PCR products amplified from plasmid pC13/F3CP5 with no insert. Each lane represents PCR product from an individual plant. Similar results were obtained in two independent experiments.

**Figure S7 BMVCP5 induced *TaPDS* silencing in both leaf and root tissues of wheat.** (**A**) *TaPDS* transcript levels in the 5^th^ systemic leaf and roots from plants infected with BMV:TaPDS (100-nt insert) or BMV:eGFP (180-nt insert) at 28 dpi, analyzed by RT-qPCR, with values normalized against relative *TaEF1α* mRNA levels. Percentage values are relative to the BMV:eGFP control. Values represent means + SD of five biological replicates, and treatment values compared (t test; **, P < 0.01). Similar results were obtained in two independent experiments. (**B**) Stability of the 100-nt *TaPDS* insert in the same tissues as in (**A**), analyzed by RT-PCR using a pair of primers flanking the cloning site (P4-F/P4-R) and the cDNA as in (**A**). Each lane represents PCR product from an individual plant. M, 1 kb plus DNA ladder; EV, a 427-bp PCR product amplified from plasmid pC13/F3CP5 with no insert.

## Supplementary Tables

**Supplementary Table S1 Disease symptoms and plant height changes of 54 wheat cultivars upon BMV infection.**

| **Wheat cultivars** | **Disease**  **symptom** | **Plant height (cm) Mock** | **Plant height (cm)**  **BMV:GFPuv** | **Plant height ratio BMV:GFPuv/Mock** |
| --- | --- | --- | --- | --- |
| Cedar | Mild | 42 | 34 | 0.81 |
| Deliver | Mild | 51 | 46 | 0.90 |
| Eltan ^a^ | Mild | 56 | 41 | 0.73 |
| Kavkaz ^a^ | Mild | 61 | 54 | 0.89 |
| NF101 | Mild | 56 | 44 | 0.79 |
| OK09520 | Mild | 41 | 35 | 0.85 |
| OK09528 | Mild | 39 | 34 | 0.87 |
| Overley | Mild | 46 | 40 | 0.87 |
| Pete | Mild | 47 | 41 | 0.87 |
| RonL | Mild | 46 | 44 | 0.96 |
| Bentley | Moderate | 43 | 37 | 0.86 |
| Denali | Moderate | 47 | 33 | 0.70 |
| Gallagher | Moderate | 45 | 33 | 0.73 |
| Iba | Moderate | 46 | 35 | 0.76 |
| Kaw 61 | Moderate | 44 | 34 | 0.77 |
| Lancer | Moderate | 45 | 35 | 0.78 |
| MIT | Moderate | 48 | 33 | 0.69 |
| MT9513 | Moderate | 46 | 38 | 0.83 |
| NF97117 | Moderate | 64.5 | 48 | 0.74 |
| OCW00S063S-1B | Moderate | 43 | 36 | 0.84 |
| OK0986050 | Moderate | 47 | 33 | 0.70 |
| Robidoux | Moderate | 52 | 43 | 0.83 |
| Shocker | Moderate | 47 | 34 | 0.72 |
| Triumph 64 | Moderate | 52 | 41 | 0.79 |
| TX04A001246 | Moderate | 47 | 34 | 0.72 |
| Apogee ^a^ | Moderate | 52 | 46 | 0.88 |
| WinTex ^a^ | Moderate | 53 | 40 | 0.75 |
| Big Maxx | Severe | 47 | 32 | 0.68 |
| Big Sky | Severe | 53 | 33 | 0.62 |
| Chancellor ^a^ | Severe | 66 | 47 | 0.71 |
| Chinese Spring | Severe | 77 | 42 | 0.55 |
| Coker 9553 | Severe | 48 | 32 | 0.67 |
| Decade | Severe | 43 | 32 | 0.74 |
| Duster | Severe | 46 | 34 | 0.74 |
| Endurance | Severe | 48.5 | 36 | 0.74 |
| Garrison | Severe | 48 | 32 | 0.67 |
| Golden Chief ^a^ | Severe | 46 | 32 | 0.70 |
| Gore | Severe | 49 | 31 | 0.63 |
| Jagger | Severe | 43 | 33 | 0.77 |
| JS-51 | Severe | 48 | 35 | 0.73 |
| Kiowa | Severe | 45 | 34 | 0.76 |
| MT0495 | Severe | 45 | 32 | 0.71 |
| NF00108 | Severe | 59 | 44 | 0.75 |
| Norkan | Severe | 42 | 30 | 0.71 |
| OK05511-Rhf2 | Severe | 47 | 35.5 | 0.76 |
| OK08328 | Severe | 46 | 30 | 0.65 |
| OK1059060 | Severe | 42 | 28.5 | 0.68 |
| OK1080029 | Severe | 51 | 35 | 0.69 |
| Rosebud | Severe | 52 | 35 | 0.67 |
| Scout 66 | Severe | 63.5 | 41 | 0.65 |
| SD00111-9 | Severe | 49 | 34 | 0.69 |
| Smokeyhill | Severe | 44 | 29 | 0.66 |
| TAM 302 | Severe | 43 | 30 | 0.70 |
| Thatcher | Severe | 69 | 48 | 0.70 |

The 1^st^ and 2^nd^ leaves of 9 day-old wheat seedlings (10 plants per cultivar) were rub-inoculated with sap from *N. benthamiana* leaves infiltrated with BMV:GFPuv or mock (no virus). Phenotypic data collected at 30 dpi.

^a^ Seeds from USDA.

Seeds for all other wheat cultivars provided by the Small Grains Laboratory (Noble Research Institute, LLC).

**Supplementary Table S2** **Primers used to construct BMVCP5 VIGS vectors**

| **Primer** | **Primer sequence (5'-3') ^a^** | **Position ^b^** | **Usage** |
| --- | --- | --- | --- |
| P1-F1 | TGTA**CCTAGG**GGAACAGTGAAGCACTTTGCA | *TaPDS-B* nt 1039-1057 | Construction of BMV:TaPDS_250_ with P1-R1 |
| P1-F2 | TGTA**CCTAGG**TGCATATGTTTTTGCAGCAC | *TaPDS-B* nt 1089-1108 | Construction of BMV:TaPDS_200_ with P1-R1 |
| P1-F3 | TGTA**CCTAGG**AAGAGTGGAGAGAGATCTCT | *TaPDS-B* nt 1139-1158 | Construction of BMV:TaPDS_150_ with P1-R1 |
| P1-F4 | TGTA**CCTAGG**GTTCCTGTCATCAATGTTCA | *TaPDS-B* nt 1189-1208 | Construction of BMV:TaPDS_100_ with P1-R1 |
| P1-R1 | TGCT**CCATGG**CTGCATAAACGCTTAAAAGTGA | *TaPDS-B* nt 1267-1288 | Paired with P1-F1 to -F4 |
| P1-R2 | TGCT**CCATGG**ACGTGTTCTTCAGTTTTCTGTCAAA | *TaPDS-B* nt 1216-1240 | Construction of BMV:TaPDS_52_ with P1-F4 |
| P2-F1 | TAAT**CCTAGG**ATTTTCCGTTGTGGAGTTGG | *TaPHO2-A* nt 1659-1678 | Paired with P2-R1 to R4 |
| P2-R1 | TGCT**CCATGG**ACAATGCAAGCCCTCAGAAG | *TaPHO2-A* nt 1891-1910 | Construction of BMV:TaPHO2_252_ with P2-F1 |
| P2-R2 | TGCT**CCATGG**TGGATATCATCTGGTAAATCATTTTGT | *TaPHO2-A* nt 1836-1862 | Construction of BMV:TaPHO2_204_ with P2-F1 |
| P2-R3 | TGCT**CCATGG**TTCAGCCAACCTCTTTTAACATTT | *TaPHO2-A* nt 1785-1808 | Construction of BMV:TaPHO2_150_ with P2-F1 |
| P2-R4 | TGCT**CCATGG**TCCTTCACAAAATGATGATCAGAA | *TaPHO2-A* nt 1749-1772 | Construction of BMV:TaPHO2_114_ with P2-F1 |
| P3-F1 | AACT**CCTAGG**GATCCGCCACAACATCGAG | *eGFP* nt 501-519 | Construction of BMV:eGFP_220_ with P3-R1 |
| P3-F2 | AACT**CCTAGG**GACCACTACCAGCAGAACA | *eGFP* nt 541-559 | Construction of BMV:eGFP_180_ with P3-R1 |
| P3-F3 | ACAT**CCTAGG**AGTCCGCCCTGAGCAAAGAC | *eGFP* nt 614-633 | Construction of BMV:eGFP_107_ with P3-R1 |
| P3-R1 | AGAA**CCATGG**TTACTTGTACAGCTCGTCCAT | *eGFP* nt 700-720 | Paired with P3-F1 to F3 |

^a^ Bold letters indicate ***Avr*II** (CCTAGG) or ***Nco*I** (CCATGG) sites; ^b^ Numbers correspond to target nucleotide positions counted from the start codon.

**Supplementary Table S3** **Primers used for RT-PCR or qPCR**

| **Primer** | **Primer sequence (5'-3')** | **Position ^a^** | **Usage** |
| --- | --- | --- | --- |
| P4-F | CTTGTGTTGCTGAGAAAC | *BMV* coat protein nt 317-334 | RT-PCR for BMV insert detection |
| P4-R | TCTTGTAAGAGGTCTGC | *BMV* coat protein nt 711-727 |  |
| P5-F | CGGTTGCTGTTGGTGTCATC | *TaEF1α-D* nt 659-678, *TaEF1α-A* nt 659-678 | qPCR as internal control |
| P5-R | TCATTGCTCGCTCGGGATAAG | *TaEF1α-D* nt 802-822, *TaEF1α-A* nt 802-822 |  |
| P6-F | ACCGAAATCATCGAAGCAAC | TaPDS-A nt 1384-1403, *TaPDS-B* nt 1384-1403, *TaPDS-D* nt 1396-1415 | qPCR of *TaPDS* homologues |
| P6-R | ACTCTGGTCAGCAGCGATTT | *TaPDS-A* nt 1436-1455, *TaPDS-B* nt 1436-1455, *TaPDS-D* nt 1448-1467 |  |
| P7-F | GATACCTCCTCCGGCTTCTT | *TaPHO2-A* nt 1237-1256, *TaPHO2-D* nt 1237-1256, *TaPHO2-B* nt 1240-1259 | qPCR of *TaPHO2* homologues |
| P7-R | ATCACACCACTAGCCCATTG | *TaPHO2-A* nt 1306-1325, *TaPHO2-D* nt 1306-1325, *TaPHO2-B* nt 1309-1328 |  |
| P8-F | GGCTGAGCAGGAAACAATTC | *TaPHO2-B* nt 1425-1444 | qPCR of *TaPHO2*-*B* |
| P8-R | CCAGTGCAGTCTTCACCAGA | *TaPHO2-B* nt 1478-1496 |  |
| P9-F | TCAGATGATGGCTGCAAGAG | *TaPHO2-A* nt 2422-2441 | qPCR of *TaPHO2-A* |
| P9-R | AGCTTCGGCAGTTGCTTG | *TaPHO2-A* nt 2472-2489 |  |
| P10-F | CTCGGCGGTGATCTCATTG | *TaPHO2-D* nt 2377-2395 | qPCR of *TaPHO2-D* |
| P10-R | AGGCGATCCCAGCTTCGC | *TaPHO2-D* nt 2504-2521 |  |
| P11-F | GGGCACAAGCTGGAGTACAAC | *eGFP* nt 415-435 | qPCR of *eGFP* |
| P11-R | TCTGCTTGTCGGCCATGATA | *eGFP* nt 456-475 |  |

^a^ Numbers correspond to target nucleotide positions counted from the start codon.
