# Supplementary figures and images for: An Efficient Brome mosaic virus-Based Gene Silencing Protocol for Hexaploid Wheat (Triticum aestivum L.)
Source: Front Plant Sci. 2021 Jun 18;12:685187. doi: 10.3389/fpls.2021.685187 (PMC8253535; doi:10.3389/fpls.2021.685187)

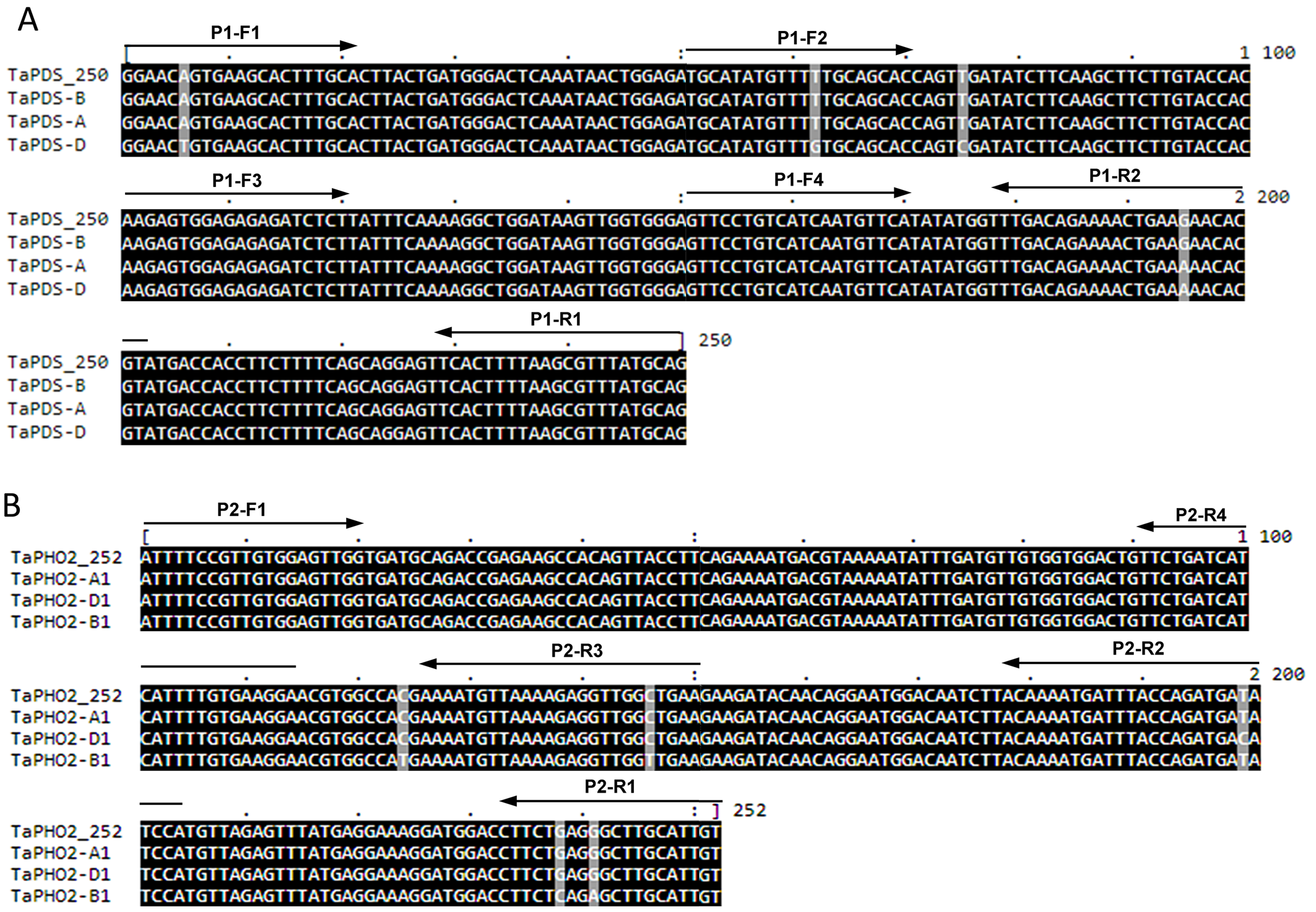

Supplement: Supplementary file 2 [file Image_1.TIF]

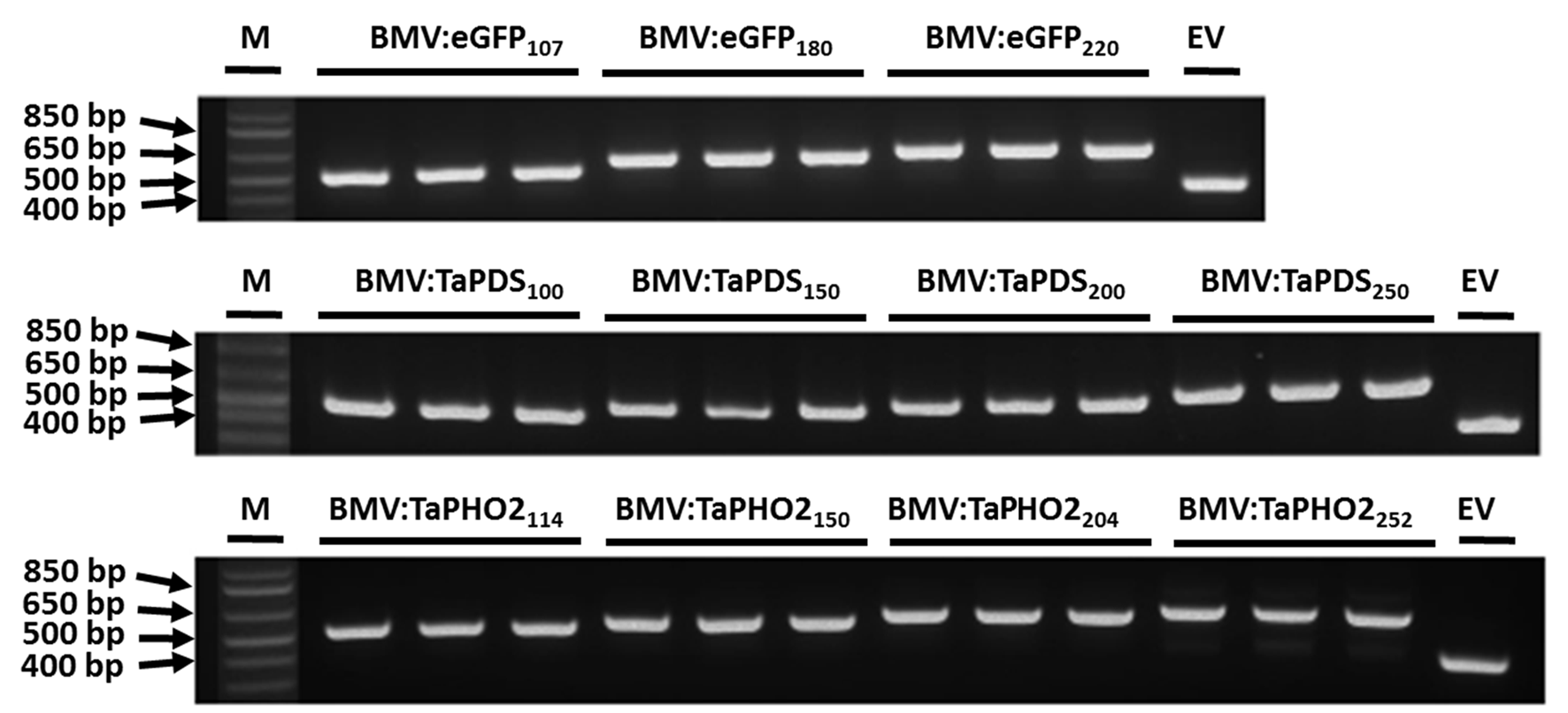

Supplement: Supplementary file 3 [file Image_2.TIF]

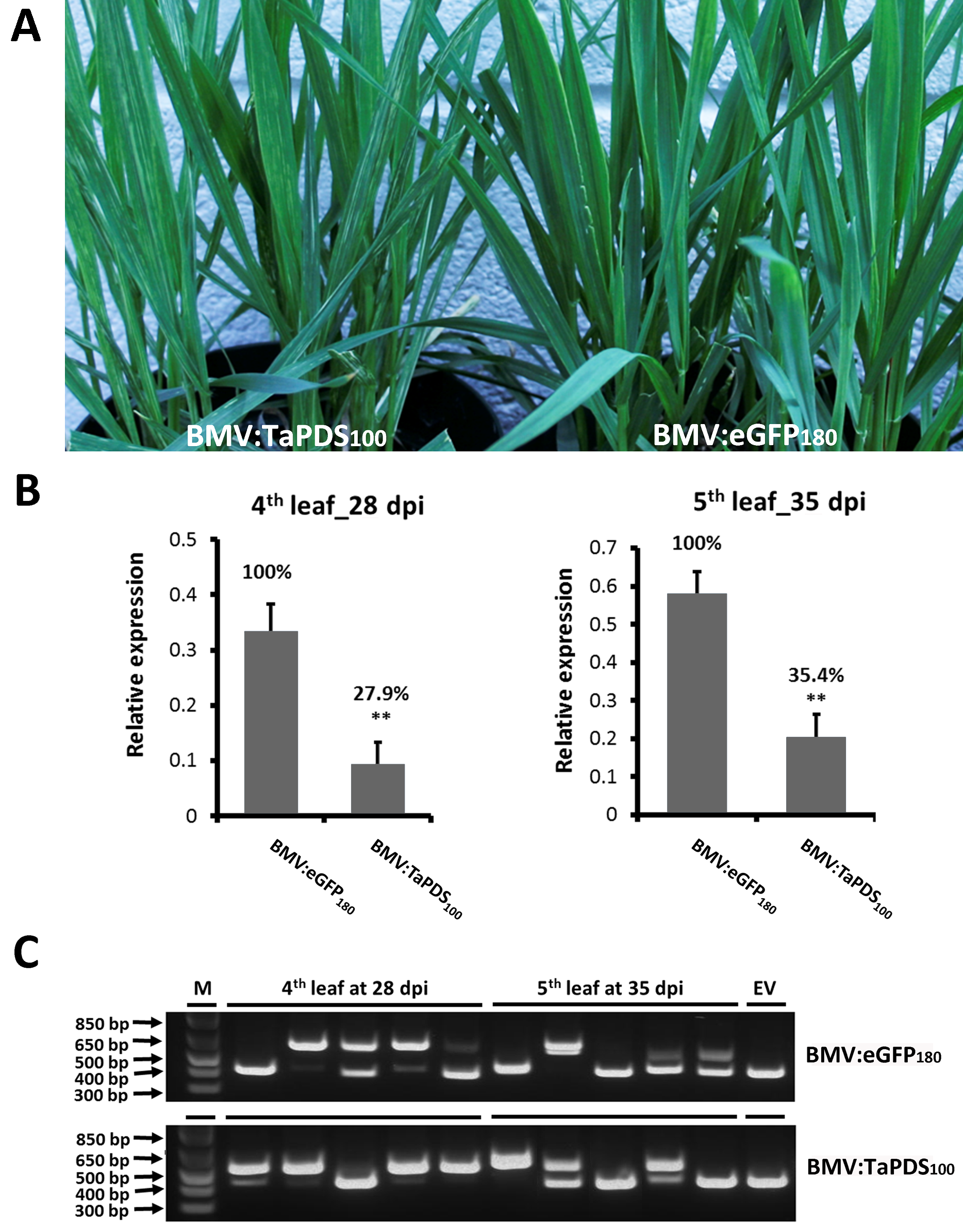

Supplement: Supplementary file 4 [file Image_3.TIF]

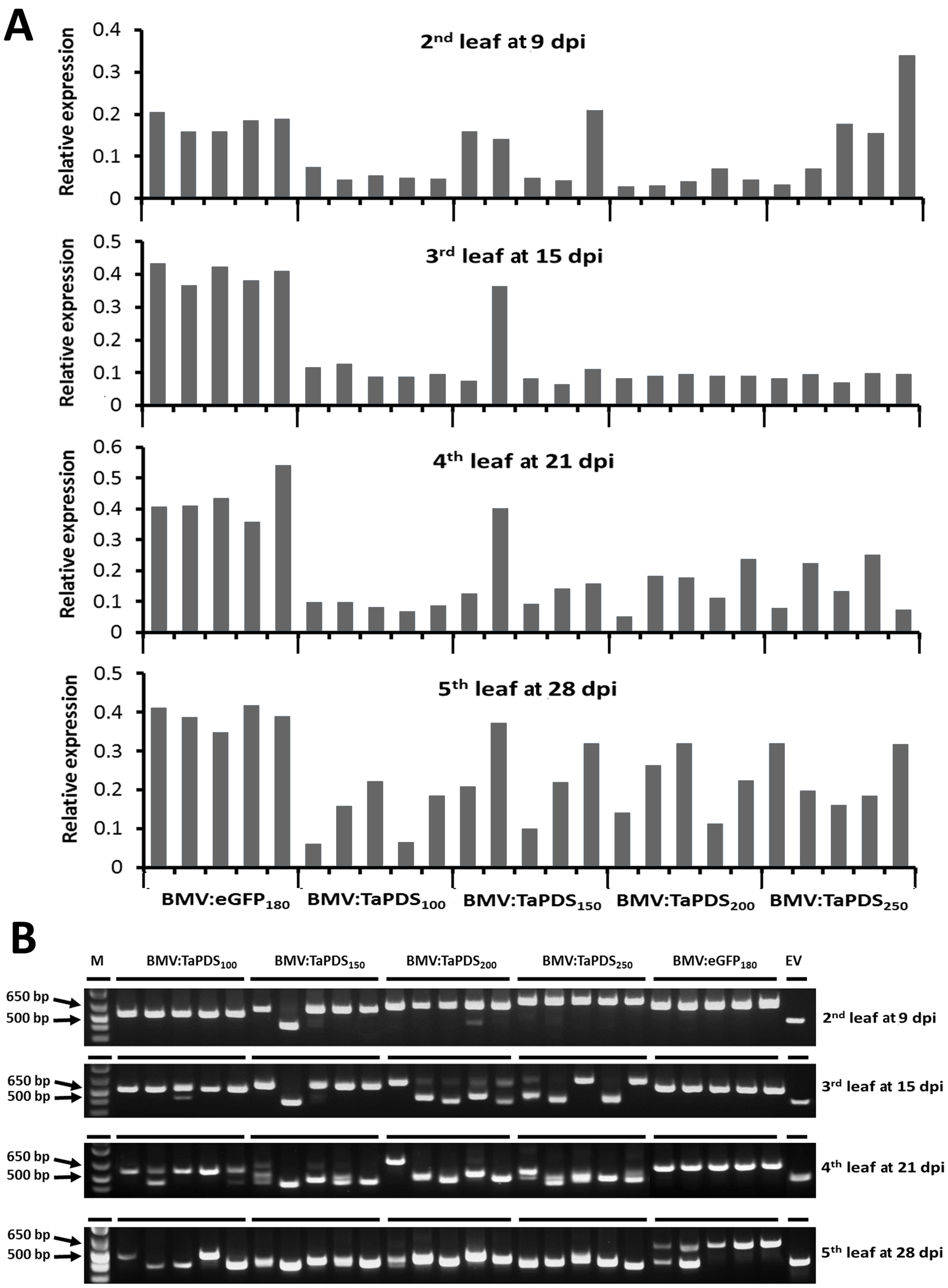

Supplement: Supplementary file 5 [file Image_4.TIF]

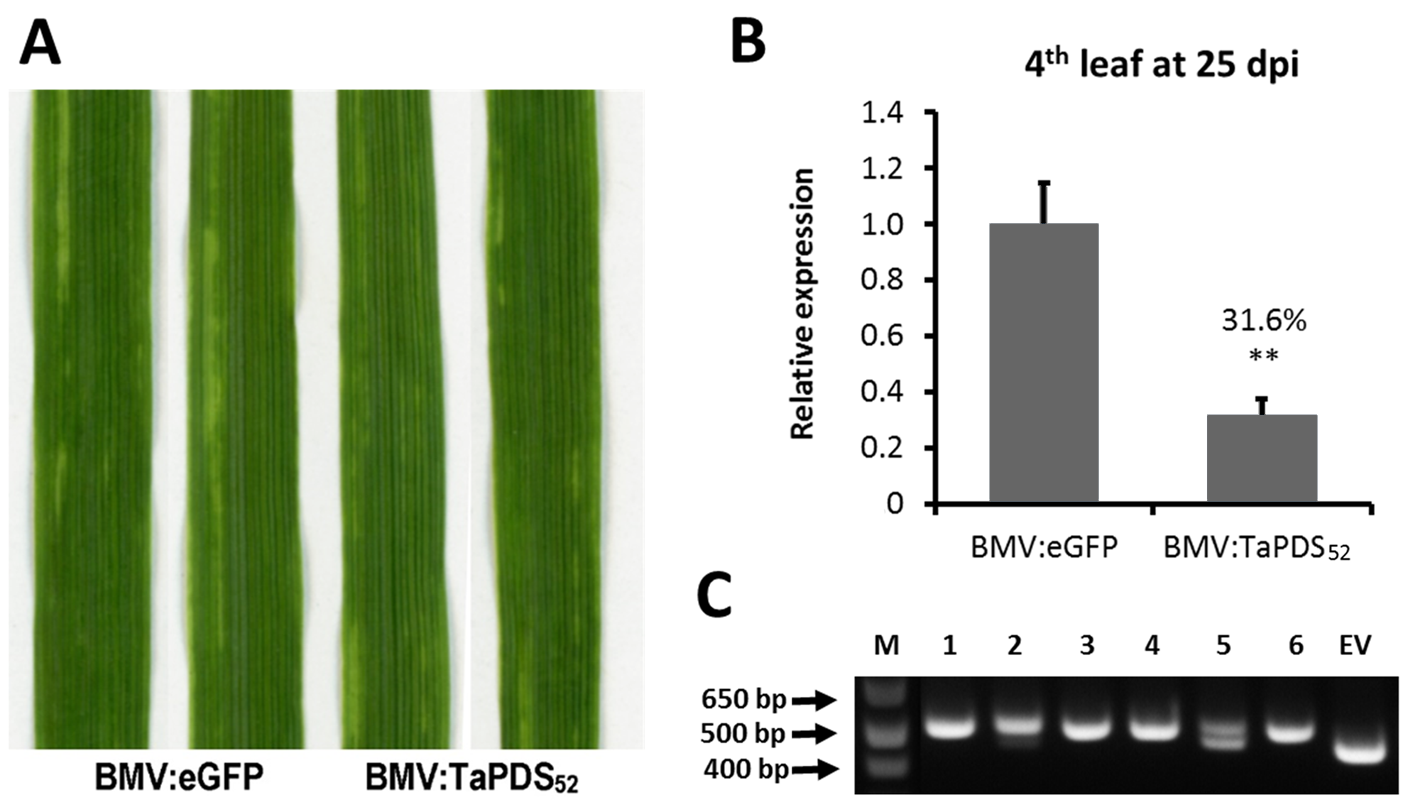

Supplement: Supplementary file 6 [file Image_5.TIF]

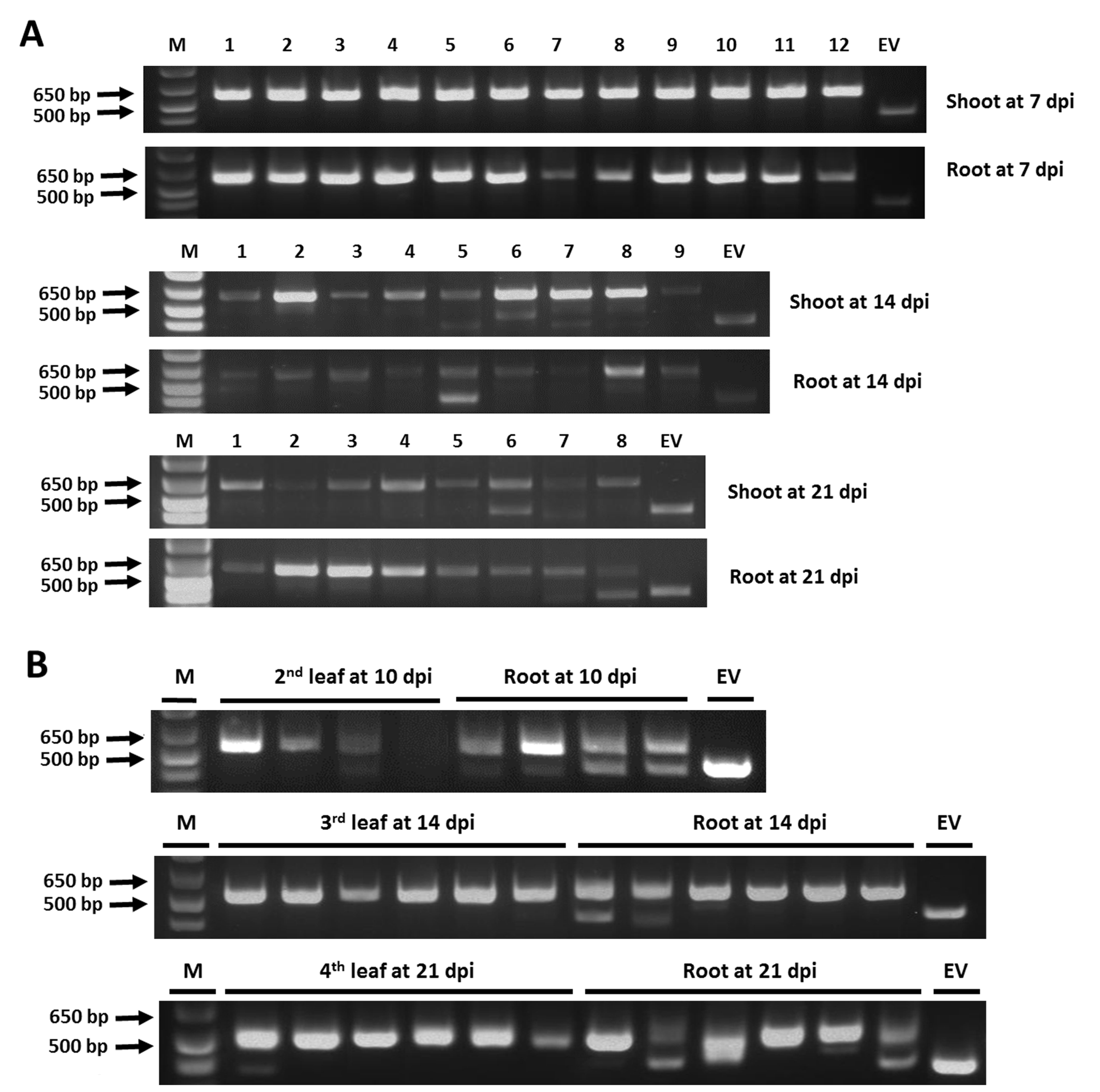

Supplement: Supplementary file 7 [file Image_6.TIF]

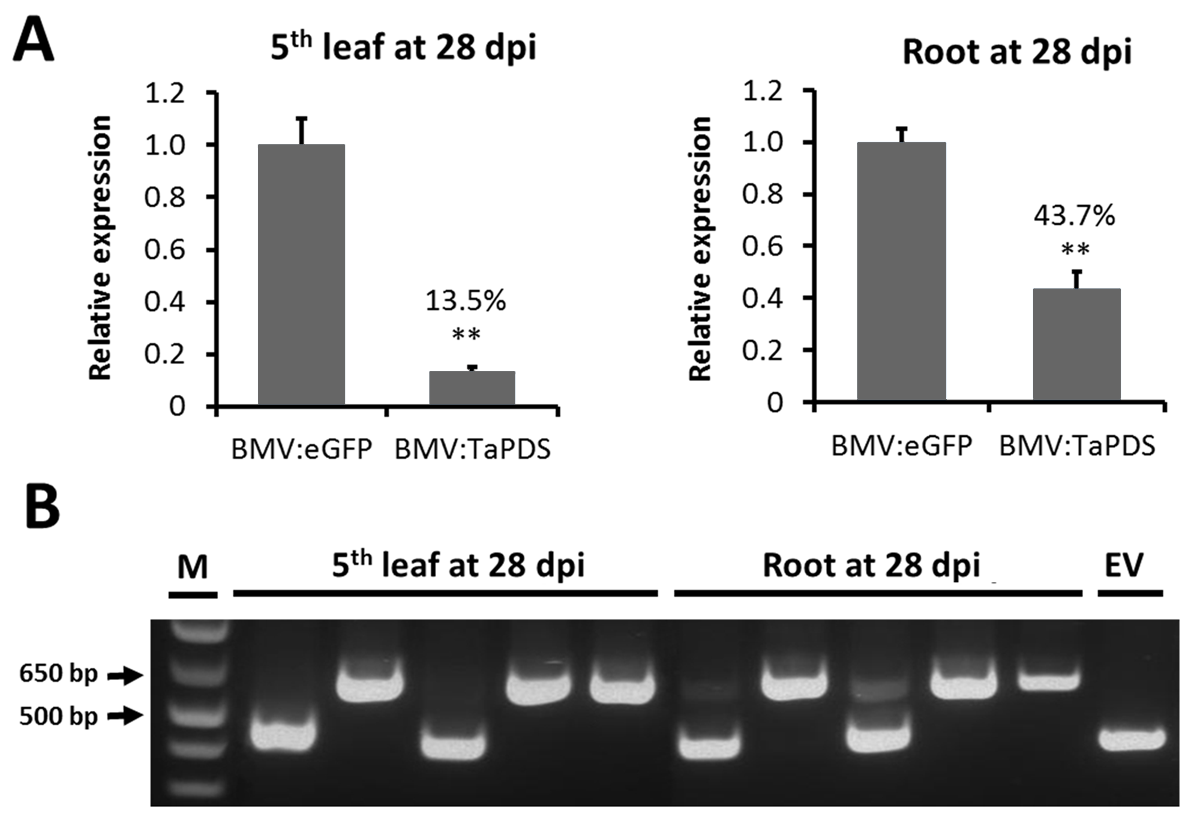

Supplement: Supplementary file 8 [file Image_7.TIF]
